# Behavior adaptation for mobile robots via semantic map compositions of constraint-based controllers

**DOI:** 10.3389/frobt.2023.917637

**Published:** 2023-08-17

**Authors:** Hao Liang Chen, Bob Hendrikx, Elena Torta, Herman Bruyninckx, René van de Molengraft

**Affiliations:** ^1^ Department of Mechanical Engineering, Eindhoven University of Technology, Eindhoven, Netherlands; ^2^ Department of Mechanical Engineering, KU Leuven, Leuven, Belgium; ^3^ Flanders Make—Leuven, Leuven, Belgium

**Keywords:** adaptable behavior, non-expert programming, world model, constraint-based control, semantic maps

## Abstract

Specifying and solving Constraint-based Optimization Problems (COP) has become a mainstream technology for advanced motion control of mobile robots. COP programming still requires expert knowledge to transform specific application context into the right configuration of the COP parameters (i.e., objective functions and constraints). The research contribution of this paper is a methodology to couple the context knowledge of application developers to the robot knowledge of control engineers, which, to our knowledge, has not yet been carried out. The former is offered a selected set of symbolic descriptions of the robots’ capabilities (its so-called “behavior semantics”) that are translated in control actions via “templates” in a “semantic map”; the latter contains the parameters that cover contextual dependencies in an application and robot vendor-independent way. The translation from semantics to control templates takes place in an “interaction layer” that contains 1) generic knowledge about robot motion capabilities (e.g., depending on the kinematic type of the robots), 2) spatial queries to extract relevant COP parameters from a semantic map (e.g., what is the impact of entering different types of “collision areas”), and 3) generic application knowledge (e.g., how the robots’ behavior is impacted by priorities, emergency, safety, and prudence). This particular design of, and interplay between, the application, interaction, and control layers provides a structured, conceptually simple approach to advance the complexity of mobile robot applications. Eventually, industry-wide cooperation between representatives of the application and control communities should result in an interaction layer with different standardized versions of semantic complexity.

## 1 Introduction

To advance the capabilities of autonomous mobile robot applications, their programming should be simplified for non-experts and involve motion control experts only when a new level of application capabilities must be realized (Wilde et al., 2018; Wilde et al., 2020; Heimann and Guhl, 2020). In addition, progress is to be made in integrating robots from several vendors into one and the same application, despite the variations in each vendor’s control software. To solve both challenges, this paper introduces a methodology that connects a low-level motion control algorithm (developed by a vendor’s control engineers) to high-level symbolic descriptions of the robots’ desired behavior (configured by application developers). The ambition is to allow an application developer to change the motion control strategy and target of the mobile robot just by changing *semantic annotations* on a geometric map. Of course, the approach is limited to motion behaviors already implemented by control engineers. For example, behaviors such as “keep enough space with walls“ and “drive forward in your traffic lane” require (re)design of the low-level motion control.

Our methodology is inspired by the traffic system, where a decoupling of responsibilities is present between the traffic regulator and the traffic participant. The traffic regulator prescribes symbolic behavioral instructions by adjusting the traffic layout with traffic signs/markings and the traffic participant interprets the composition of those traffic signs/markings and executes its motion accordingly. This is realizable as there is common knowledge between the two entities in the form of traffic signs/markings. Similarly, we introduce common knowledge components between the control engineer and the application developer. These knowledge components are symbolic descriptions of 1) the generic behavior that needs to be executed, 2) the geometric area in which this behavior needs to be executed, and 3) the intention for executing the behavior. The application developer uses these descriptions to design new behavioral rules, for example, 1) execute a stopping motion, 2) in front of the red stop light, and 3) with an intention to prevent future collisions. The control engineer translates these symbolic descriptions into a Constraint-based Optimization Problem (COP) that can be solved by robot motion control. A proper design of these common knowledge components allows decoupling of responsibilities between the application developer and the control engineer.

Section 2 gives an overview of related studies. Section 3 describes our first contribution that translates the principles of the traffic system into our three-layered methodology. Section 4 describes our second contribution in extracting robot-executable commands from the methodology. Section 5 describes our third and final contribution in which the applicability of the methodology is shown in simulation and real-world experiments. Our methodology trades potential in optimality in favor for facilitated programming by non-experts. Hence, no claims are made regarding superiority in quantitative measures as time or distance minimization. Control engineers on their own will most likely design a more optimal COP by specifically parameterizing it to the used COP solver and environment (Mercy et al., 2018). Section 6 provides a conclusion and discussion of the methodology.

## 2 Related work

Core behaviors of mobile robots are to reach a target in a map (Moravec and Elfes, 1985), not to collide with occupied areas while driving to the target (Elfes, 1989; LaValle and Kuffner Jr, 2001; Marder-Eppstein et al., 2010; Mercy et al., 2018; Macenski et al., 2020), and to adapt the driving behavior to safety and inter-robot coordination constraints (Wilde et al., 2018). A real-world example of such constraints is the traffic system: traffic signs and markings regulate, warn, or guide all traffic participants (DoT, 2009). It is the traffic participants’ responsibility to adapt their actual driving to a variety of areas with different traffic conditions. For autonomous robots, such adaptations have been introduced in several ways (Kuipers, 2000): adapting to geometric characteristics of the environment (Caloud et al., 1990; Asama et al., 1991; Kato et al., 1992), relating behavioral restrictions to areas for the coordination of multi-robot passages, e.g., “not to enter an intersection if something else is present” (Kim et al., 2016; Wilde et al., 2018; Ravankar et al., 2019; Wilde et al., 2020), and adding areas that increase cost functions in path planners (Hart et al., 1968). In principle, all of that mentioned above can be incorporated into a COP, more in particular, of the Model Predictive Control (MPC) variety (Mayne et al., 2000; Mercy et al., 2018).

### 2.1 Semantic maps

A *semantic map* (Galindo et al., 2005; Nüchter and Hertzberg, 2008; Deeken et al., 2015; Kostavelis et al., 2016; Ruiz-Sarmiento et al., 2017; Deeken et al., 2018; Varanka and Usery, 2018; Joo et al., 2022) *annotates* points and areas in a geometric map with labels that represent a *symbolic* relation between the annotation and the envisaged motion behavior adaptation in the annotated area (Galindo et al., 2005; Nüchter and Hertzberg, 2008; Deeken et al., 2015; Kostavelis et al., 2016; Ruiz-Sarmiento et al., 2017; Deeken et al., 2018; Varanka and Usery, 2018; Joo et al., 2022). Well-chosen labels facilitate an application developer’s job “to program” robots, or rather, *to configure* their behavior. In addition to the human behavior configuration, action planners have been developed, such as STRIPS (Dornhege et al., 2010; Kuipers et al., 2017) or PDDL (Fox and Long, 2003; Gerevini et al., 2009; Joo et al., 2022). These solve a *constraint satisfaction problem*, where the constraints are Boolean *pre-* and *post*-conditions on the status of the world. For example, “to travel to the fridge, one first needs to enter the kitchen.” The MPC approach of COPs adds *per* conditions in continuous time and space, that is, (in)equality constraints that have to be *satisfied*, or objective functions that have to be *optimized*, *during* the execution of a motion. In a semantic map context, both boolean and continuous constraints are complementary. For example, control instructions are related to geometric areas in the semantic map (Caloud et al., 1990; Asama et al., 1991; Kato et al., 1992).

### 2.2 Continuous parts of COPs

“Objective functions” in the path planning literature are typically referred to as cost criteria, where the objective is to minimize a cost criterion such as the traveled distance. In the context of this paper, cost criteria are related to entering, traversing, and/or leaving geometric areas in the semantic map. The simplest costs come from distances between “unoccupied areas” and the “target” (Moravec and Elfes, 1985) and relative spatial locations of “occupied areas” (Elfes, 1989; LaValle and Kuffner Jr, 2001; Marder-Eppstein et al., 2010; Macenski et al., 2020). These simple cases suffice for tasks whose sole objective is to navigate to the target without collisions. The authors consider the terminology of “objective functions” and “cost criteria” as synonyms, and for consistency, we will use the term “objective functions” throughout the paper.

Path geometries can be adapted to a semantic context by 1) *changing the weight* of one objective function in the whole objective functions, 2) *adding new objective functions* to semantic areas, 3) *semantically annotating* more areas in the semantic map, or 4) adding *constraints* between the properties in the geometric map and/or the semantic relations. Kim et al. (2016) added region with velocity constraints (RVCs) on areas that are difficult to traverse, e.g., areas near water or with a muddy surface. Pierson et al. (2018) added an objective function to the distance between cars on a highway with multiple lanes to facilitate safe lane transitions. Sünderhauf et al. (2016) added objective functions to the traversal of office and corridor areas to allow the avoidance of busy office areas at the expense of longer path lengths. Similarly, Chen et al. (2022) added an objective function to the type of surface; e.g., it is more important not to traverse “areas with green plants” than “normal ground surfaces.” Mainprice et al. (2011) added terms related to human comfort to, for example, allow the robot to remain in the field of view of humans while navigating. Ravankar et al. (2019) introduced inconvenient area costs. All objective functions can end up as a *weighted sum* possibly extended with a *prioritization* mechanism. Campos et al. (2019) added pose constraints with respect to humans, distance constraints with respect to humans, and distance constraints with respect to the wall. Shi et al. (2008) added velocity constraints to let a robot pass a human in a safe manner.

To the best of the authors’ knowledge, none of the previously mentioned state-of-the-art publications provides *explicit formal models* of the objective function or constraint knowledge they introduce in the COP specification. Hence, it is impossible for a robot to formulate (or, rather, to (re)configure) such a COP *itself* and to adapt its motion to the information provided by the semantic map. For example, knowledge on why a certain objective function is more important than another and why the difference in the importance between two objective functions must be exactly the specific value and nothing else. Seminal work in such composition is the subsumption architecture of Brooks (1986), but without explicit and formal design knowledge.

### 2.3 Discrete parts of COPs

The Task and Motion Planning literature introduces first-order logical relations between symbolically represented actions to deduce the proper sequence of actions to fulfill a task by means of STRIPS, PDDL, or PROLOG engines (Fox and Long, 2003; Gerevini et al., 2009; Dornhege et al., 2010; Waibel et al., 2011; Kuipers et al., 2017; Tenorth and Beetz, 2017; Beetz et al., 2018; Faroni et al., 2020; Joo et al., 2022). We recall that it is not an objective of this paper to contribute to the improvements of such reasoning approaches and engines. The first-order relations can be of various types:

• *Pre-conditions:* to restrict the (de)activation of actions when the world is in a particular state
• *Post-conditions:* on the effects that executed actions have on changes in the state of the world
• *References to algorithms* that need to be executed during the solving of the COP and/or the perception required to update the world model

For example, a pre-condition of a “move” action is that the robot has motion-allowing components such as wheels and motors; a post-condition is a sufficiently large change in the position of the robot.

It is not always necessary to let the reasoning be carried out by the robot, *at runtime*, because one can *store* the outcome of *offline*-executed reasoning into geometric areas of the semantic map and trigger its use as soon as the robot enters or leaves the areas. For example, the abovementioned velocity constraints of Kim et al. (2016) are applied as soon as the robot is contained within an RVC area. Similarly, with the behavior adaptation policies in the work of Caloud et al. (1990), Asama et al. (1991), and Kato et al.( 1992), “a robot must not enter a crossroad if another robot is present there,” or “if another robot is approaching, then avoid it to the right.” Kuipers (2000) introduced explicit control specifications related to spatial characteristics in the geometric base map and stored them in the semantic map.

## 3 Methodology

The core technical contribution of this paper is the three-layered structure, *application*, *interaction*, and *control*. The major motivation is that the application layer helps non-experts to program mobile robots because its interface reflects the *traffic system*, with its widely understood semantics of traffic *code*, *signs*, and *markers*.

### 3.1 Inspiration: the traffic system

The traffic system is a successful realization of a decoupling of responsibilities between the traffic *regulator*, who “annotates” areas in traffic with behavioral *constraints*, and a traffic *participant*, who realizes driving motion behaviors that satisfy those constraints. The semantics of traffic are provided in terms of *Traffic Control Devices* (TCDs), defined in standardization documents such as the *Manual on Uniform Traffic Control Devices* (DoT, 2009).

Definition 1: ***TCDs*** *shall be defined as all signs, signals, markings, and other devices used to regulate, warn, or guide traffic, placed on, over, or adjacent to a street, highway, pedestrian facility, bikeway, or private road open to public travel by the authority of a public agency or official having jurisdiction, or, in the case of a private road, by the authority of the private owner or private official having jurisdiction.*

In practice, a TCD is the combination of 1) a constraint on the position and velocity of traffic participants and 2) a geometric area in the world where the constraint holds. For example, a “trucks use right lane”-sign obliges trucks to enter only geometric areas that represent right lanes, and a “yield for pedestrians”-sign obliges traffic participants to stop in front of a pedestrian crossing area. TCDs that are laid out according to the *best practices* of traffic system design are straightforward to compose; e.g., the composition of both mentioned signs remains semantically unambiguous for traffic participants. From a robotics system perspective, this compositional nature of TCDs is a perfect fit for motion control specified as COPs. Another excellent property of the traffic system is its unambiguous *priority ordering* of composed TCDs, including the enumeration of *situational conditions* in which the *intent* of (some) constraints in TCDs can be violated.

### 3.2 Entities of the methodology

**FIGURE 1:**
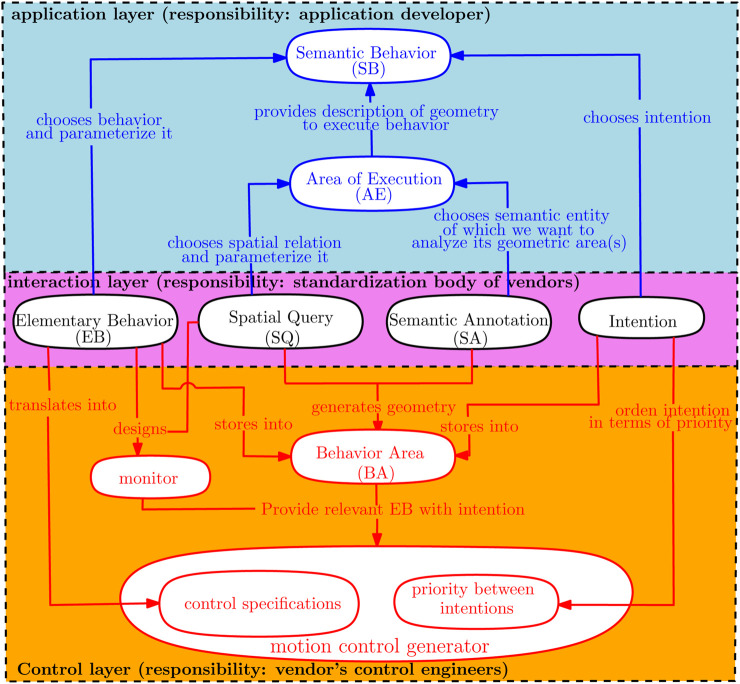
Overview of the responsibilities of application developers (in blue) and control engineers (in red). The *interaction layer* represents the symbolic knowledge that application developers and control engineers need to share. The *application* layer allows the application developers to program their robots via symbolically described behavior and areas. The *control* layer translates symbolic behavior descriptions into parameters in 1) motion control algorithms and 2) geometric maps.

Figure 1 sketches the methodology as a three-layered structure with relations clarifying the decoupled responsibilities between the application developer (blue arrows) and the control engineer (red arrows). The *interaction layer* in the middle represents the symbolic interface to generate a model of *TCDs*, as discussed previously. Ideally speaking, this interface should (eventually) be standardized by a “Foundation” that represents all mobile robot vendors.

Definition 2: ***Elementary Behaviors*** (***EBs***) *are symbolic labels that describe simple restrictions on a mobile robot’s position and velocity, independent of the hardware characteristics of individual robots.*

This paper introduces four EBs: “stop in this area,” “drive forward with a certain speed,” “avoid this area,” and “do not enter this area.”

Definition 3: ***Semantic Annotations*** (***SAs***) *to areas on a geometric map represent the human interpretation of that area.*

In this paper, a **semantic map** is a geometric map with semantic annotations.

Definition 4: ***Spatial Queries*** (***SQs***) (*Sack and Urrutia, 1999*) *are queries in a spatial database that can be answered on the basis of geometric information only, that is, the spatial position and extent of the entities involved.*

In this work, the spatial database contains the geometric areas of the geometric map, and the spatial queries retrieve geometric areas, and two types of spatial queries are considered: 1) Filtering Spatial Queries (FSQs), which yield a list of geometric areas filtered by semantic annotation, and 2) Transformative Spatial Queries (TSQs), which create new geometric areas by transformations on some SAs. An example of a FSQ is to retrieve all geometric areas of chairs that are contained within the kitchen, formulated as “ContainedIn(chairs, kitchen).” An example of a TSQ is to generate a 5 cm area around those chairs, formulated as “AreaAround (ContainedIn(chairs, kitchen), [buffer = 5 cm]).”

Definition 5: ***Intentions*** *are the reasons why application developers add an EB in their application.*

Definition 6: ***Priority*** *between intentions is an ordered list of intentions.*

This work considers the intentions of “Progress” (to guide the robot toward task completion), “Safety” (to warn robots for collision risks), “NoDamage” (to prevent immediate collisions), and “CompleteTask” (to indicate that a task is completed). Priorities are used in the COP generator to deduce importance between all involved EBs.

Definition 7: ***Area of Execution*** (***AE***) *is a symbolic description of the geometric area(s) which relate to the execution of EBs.*

Definition 8: ***Semantic Behavior*** (***SB***) *is a symbolic description of mobile robot behavior related to semantic annotations on a semantic map, by identifying 1*) *the EBs to execute, 2*) *the AE in which this behavior applies, and 3*) *the intention behind each SB.*

Definition 9: ***Behavior Area*** (***BA***) *in a geometric map contains a list of EBs, including their intentions.*

Definition 10: ***Behavior map*** *is a geometric map with BAs.*

In this work, BAs include the “stop area,” “drive forward area,” “avoid area,” and “no-enter area.”

The *application layer* in Figure 1 contains the application developers’ SBs. It consists of the type of SQs that need to be performed and also the types of SAs that form the input to retrieve the geometric area(s).

The *control layer* represents the responsibilities of the control engineer. As the SBs are solely a function of the components in the *interaction* layer, a control engineer can focus itself on translating these components.

Definition 11: ***Control specification*** *is a translation of an EB into parameters of low-level motion control algorithms.*

Definition 12: ***Monitor*** *is a condition that represents when an EB should be executed.*

In this work, monitors indicate when EBs should be executed as SQs on the robot and the AE it finds itself in. For example, a *stop behavior* is executed whenever the robot has fully entered the corresponding stop area. BAs are generated from the SB by executing the queries in the AE, and symbolic EB knowledge and intention are stored within that area. A behavior map is created from these BAs. The control engineer ranks the intentions in terms of priority specified by the application developers, such that the robot can determine what selection of behaviors are most important at any given time. With the monitors and the behavior map in place, a mobile robot queries its behavior map for the currently relevant EBs and provides them to the motion control generator. In this work, the generation results in a COP, that is, an objective function and (in)equality constraint(s). Obviously, COPs are only one of many possible types of motion controllers.

### 3.3 Impact of the methodology

The impact of the presented methodology comes from the structured decoupling between the application layer and the control layer, via a (standardized) interaction layer, that allows the two layers to be independently developed. The methodology allows the application developers “to (re)program” the behavior of their robots in three complementary ways, by adjusting the relation between an EB and a SA, the area in which EBs are effective, and/or the intention of SBs. We thus transfer the complexity of behavior design to the high-level application layer, while the “lower” interaction and control layer can be described in a generic manner with, e.g., EBs. The methodology does introduce a dependency, and that is on the particular *version* of the interaction layer semantics. Motion control engineers only need to add new motion control functionality when semantics is updated, and that happens when application developers have identified the need for one or more fundamentally new SBs.

### 3.4 Example behavior map

**FIGURE 2:**
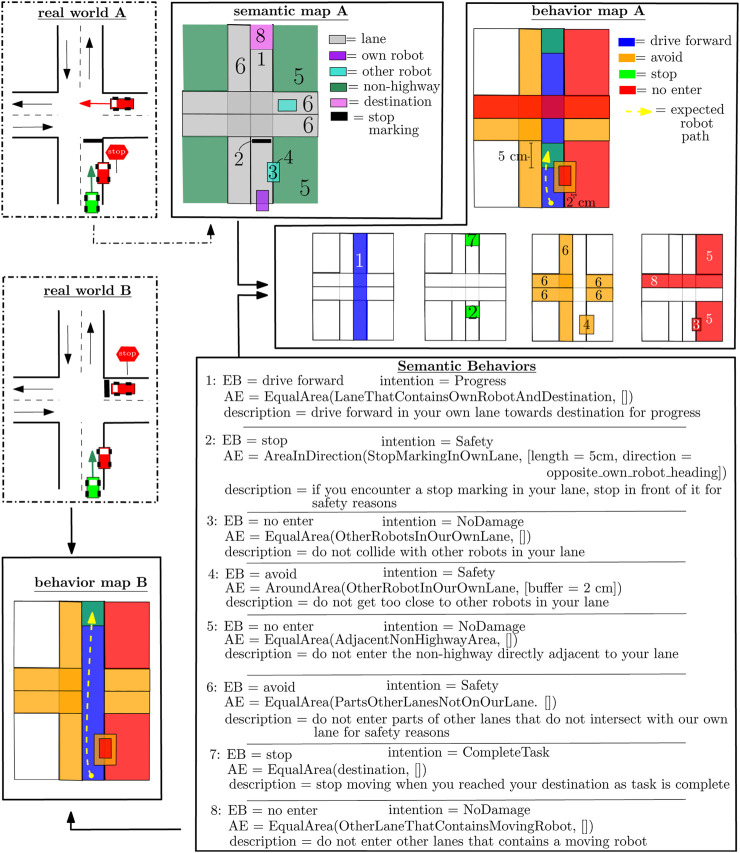
Example of two different behavioral maps on the same geometric map. The numbers on semantic map A and behavior map A represent the related SB. The BAs can overlay. FSQs are described as a concatenated SA for readability, e.g., “LaneThatContainsOwnRobotAndDestination,” formally written as “Contains (Contains (lane, robot), destination).”

Figure 2 depicts two two-lane crossing scenarios where the difference is observed by the placement of the stop sign/marking. This single difference results in differing behavior maps. The SAs on the semantic map in combination with the SBs result in a behavior map. Each SB is described by a combination of EB, AE, and intention. A description is provided of the interpretation of the SB. The AE is described as a TSQ executed on some semantic area with some parameterization. This semantic area is formally described as a FSQ, but for simplicity, we have described it with a single statement. For example, the statement “StopMarkingInOwnLane” is formally described as the FSQ “Contains (Contains (Lane, Robot), StopMarking).” The TSQ “EqualArea” represents that the geometric area of a BA is equal to the geometric area of the underlying semantic area. The TSQ “AroundArea” generates a new geometric area around an existing geometric area, and “AreaInDirection” generates a geometric area in a certain direction with an existing geometric area as a starting point.

The behavior map is a composition of BAs, where behavior map “A” explicitly shows the separate maps containing specific types of BA. This clarifies that the methodology allows BAs to be stacked on each other such that new BAs can be independently introduced via independently designed SBs. This independency simplifies the design of behavioral rules for the application developer. In behavior map “A,” all SBs are relevant. In behavior map “B,” SB 2 is not relevant as the stop marking is not in our robot lane anymore. SB 8 is not relevant as the other robots do not move anymore (due to the displaced stop sign). The resulting BAs are not added to the behavior map, and subsequently, the behavior map changes.

## 4 Implementation

The contribution of this section is the realization of the methodology in a robot program. The first part pertains to the application layer of formulating SBs, and the second part to the control layer of generating the behavior map and COP.

### 4.1 Application layer

**FIGURE 3:**
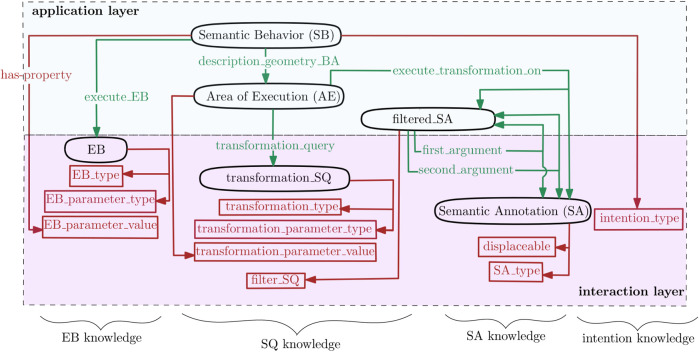
Property graph entities for the symbolic representation of SBs and AEs: black ovals represent *nodes*, green arrows represent *relations* between nodes, and brown squares represent *properties* of nodes.

An application developer designs SBs as compositions of symbolic components in the *interaction* layer (Figure 1). Figure 3 visualizes these relations in the form of a property graph, that is, a graph in which nodes and edges are labeled and provided with properties. This paper uses JSON^1^ as formal encoding:

JSON_entity = {
properties: {property_1: property_1_value, property_2: property_2_value}, relations: {relation_1: JSON_entity_2, relation_2: JSON_entity_3}
}

More concretely, the SB “drive forward in the direction [1,0] in the lane that contains the robot and destination with speed limits of 2 m/s to make progress” is represented as a composition of EBs and AEs:

SB_drive_forward_in_traffic_lane_to_destination = {
properties: {EB_parameter_value: [2, 40, [1,0]], intention_type: Progress},
relations: {execute_EB: EB_drive, description_geometry_BA: AE_traffic_lane}
}

#### 4.1.1 Elementary behavior representation

Figure 3 (left) represents the property graph of an SB and its EB entities as the relation execute_EB and the property EB_parameter_value, respectively. The representation of the EBs (Section 3.2) is in itself is a property graph:

EB_stop = {
properties: {EB_type: stop, EB_parameter_type: []},
relations: {}
},
EB_drive = {
properties: {EB_type: drive forward,
EB_parameter_type: [’translational speed limit’,
’rotational speed limit’,
’direction array’]},
relations: {}
},
EB_avoid = {
properties: {EB_type: avoid,
EB_parameter_type: [’translational speed limit’,
’rotational speed limit’]},
relations: {}
},
EB_no_enter = {
properties: {EB_type: no_enter, EB_parameter_type: []},
relations: {}
}

The property EB_parameter_type is configured via property EB_parameter_value. For example, the SB mentioned above would configure the translational speed limit to be 2 m/s, the rotational speed limit to be 40 deg/s, and the drive forward direction array to be [1,0]. The configurability of speed limits and direction arrays makes sense in the context of our traffic system inspiration, where the speed limit signs and white directional arrays are omnipresent along roads. For the avoid behavior, it makes sense to configure a lower speed limit as this behavior is typically executed in risky situations, such as avoiding other cars.

#### 4.1.2 Area of Execution representation

Figure 3 (middle) represents the property graph of an AE entity as a relation between SQs and (the geometric area of) SAs. For each AE, the application developer chooses 1) the type of TSQ, 2) the geometric area on which the TSQ should be executed, and 3) the TSQ parametrization. This choice involves, respectively, relations transformation_query and execute_transformation_on and property transformation_parameter_value. The AE also relates to the SB mentioned above via relation description_geometry_BA as follows:

AE_traffic_lane = {
properties: {transformation_parameter_type: []},
relations: {transformation_query: SQ_EqualArea, execute_transformation_on: filtered_SA_1}
}

#### 4.1.3 Spatial query representation

**FIGURE 4:**
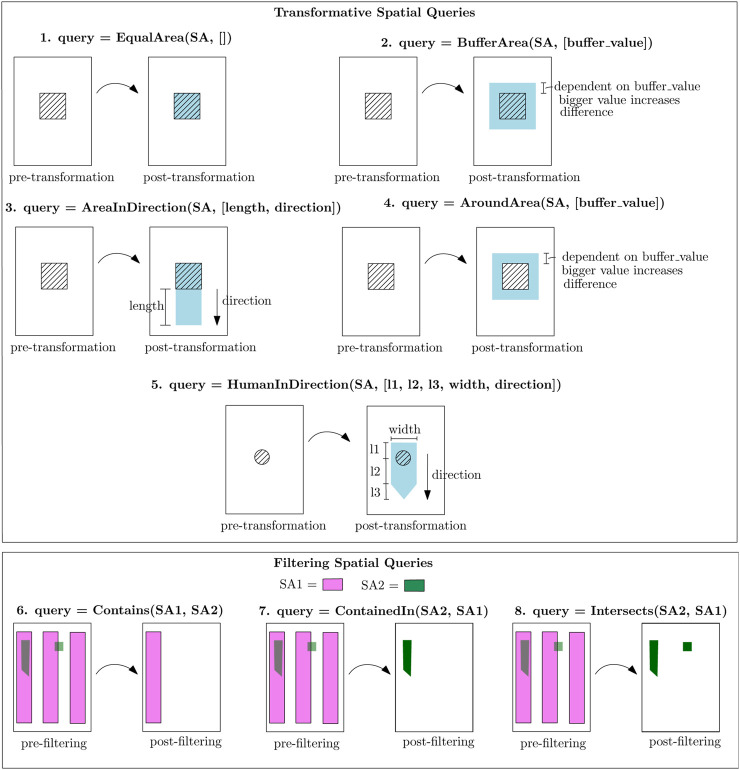
Spatial queries developed in this paper. Upper part: transformative SQs with a pre- and post-transformation result. Lower part: filtering SQs with a pre- and post-filtering result; the first argument represents the area that is filtered by checking the spatial relation with respect to the second argument. The HumanInDirection shape is inspired from human comfort analysis (Neggers et al., 2022).

Figure 4 visualizes the spatial queries introduced in this paper. TSQs are referred to as “TSQ (SA, [parameterization]),” where the argument “SA” implies the geometric area of the SA that is retrieved from a semantic map. For example,

SQ_EqualArea = {
properties: {transformation_type: EqualArea, transformation_parameter_type: []},
relations: {}
}
SQ_BufferArea = {
properties: {transformation_type: BufferArea,
transformation_parameter_type: [buffer_value]},
relations: {}
}

Application developers configure the parameters via the SB property transformation_parameter_value in a similar fashion as for EB parameters.

FSQs are referred to as “FSQ (SA_1, SA_2),” where the argument “SA_1” is the geometric areas of the SA that need to be filtered, and argument “‘SA_2” is the areas of the SA which are used to check spatial relations such as “Contains.” The knowledge schema of Figure 3 refers to the output of an FSQ as the entity “filtered_SA”; it contains the type of FSQ to execute and its first and second arguments “SA_1” and “SA_2”:

filtered_SA_1 = {
properties: {filter_SQ: Contains},
relations: {first_argument: filtered_SA_2, second_argument: SA_robot
}
}
filtered_SA_2 = {
properties: {filter_SQ: Contains},
relations: {first_argument: SA_lane, second_argument: SA_destination}
}
SA_robot = {
properties: {SA_type: Robot, displaceable: true},
relations: {}
}
SA_destination = {
properties: {SA_type: destination, displaceable: false},
relations: {}
}
SA_lane = {
properties: {SA_type: lane, displaceable: false},
relations: {}
}

The “SA_1” or “SA_2″ argument can refer to another “filtered_SA” entity, as seen in “filtered_SA_1” previously. This allows the description of more complex semantic areas, e.g., “the lane that contains both the destination and the robot” given in a nested FSQ expression as “Contains (Contains (lane, destination), robot).” The application developer can then choose to execute the TSQ on an unfiltered or filtered SA expression. The property displaceable in the SA entity indicates whether the entity can be moved or not (Section 4.2.1).

### 4.2 Control layer

The control layer is responsible for the following operations:

1) Generating BAs from SB entities
2) Determining relevant BAs from the (perception) monitor
3) Creating control specifications according to the relevant BAs
4) Generating the COP from the control specifications

**FIGURE 5:**
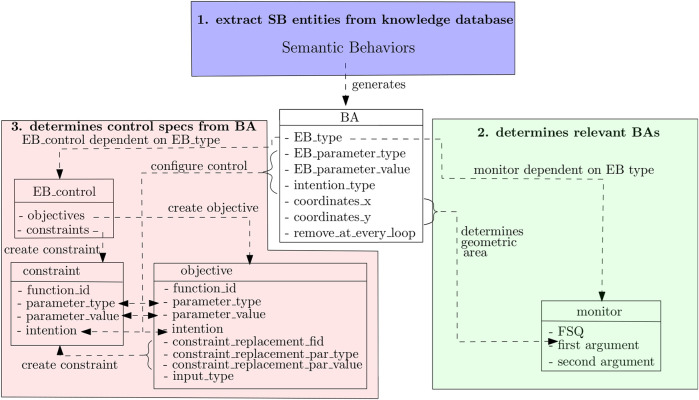
Data structures of the BA, monitor, and control specifications. These data structures are related to each other via dashed arrows connecting their members.

Figure 5 visualizes the involved data structures as rectangles and relations between them as dashed arrows.

#### 4.2.1 Generating and updating the behavior map.

**FIGURE 6:**
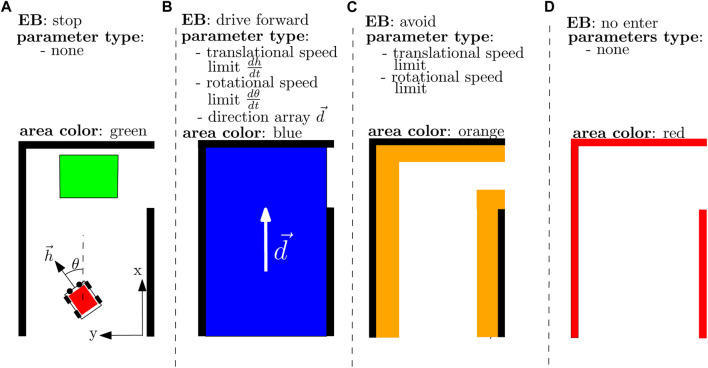
Description of the EBs with a visualization of the corresponding BA in the behavior map. In **(A),** there is a green *stop* area; in **(B),** there is a blue *drive forward* area with the white arrow indicating the *drive forward* direction; in **(C)** there is an orange *avoid* area; and in **(D),** there is a red *no-enter* area.

BAs are generated from the SBs. Figure 6 depicts the BA types used in this paper. The SB entity of the previous subsection may result in the following BA data structure:

BA = {
EB_type: drive
EB_parameter_type: [’translational speed limit’, ’rotational speed limit’, ’direction’],
EB_parameter_value: [2, 40, [1,0]],
intention_type: ’Progress’,
coordinates_x: [0, 10, 10, 0],
coordinates_y: [0, 0, 6, 6]
remove_at_every_loop: false
}.

Its first three members can be retrieved from the EB relations and properties, and its fourth member from the intention property. The last three members can be retrieved by solving the connected AE entity, where the first two store the geometric area and the last one indicates whether the BA needs to be removed at every program loop. This is necessary whenever the BA is generated from SBs that involve displaceable objects. For example, the BAs in Figure 2 that result from SB3 and SB4 where we want the BA to move whenever the other robot moves.

Definition 13: ***Continuous semantic behaviors*** *are SBs that apply the TSQ on SAs which are deemed displaceable, that is, whose position in the semantic map can vary over time. The resulting BAs in the behavior map then also vary over time.*

The continuous or non-continuous label allows the robot to only recalculate those parts of the behavior map that can change. Algorithm 1 shows the procedure to categorize SBs from the connected AE entities. Line 4 retrieves the AE entity from the SB entity and determines the entity to apply the TSQ. If this TSQ is directly applied on an SA entity at line 5, then we deem the SB continuous if the displaceable property is true at lines 6–10. If this TSQ is applied first on a filtered_SA entity, then we keep on querying the first_argument relation until an SA entity is retrieved at lines 12–14. For example, for FSQ “Contains (Contains (lane, destination), robot),” the first_argument relation that yields an SA entity is “lane.” It should be noted that the eventual retrieved geometric area is of this type SA. We can then deem an SB continuous in a similar manner as mentioned above at lines 16–20.

Algorithm 1 Determining whether SB is (non)continuous by querying the knowledge graph

1: **procedure** DetermineContinuousSB
2:   - Continuous_SB = [], Non-Continuous_SB= []
3:   **for** each SB **do**
4:     - query AE entity from SB [relations][description_geometry_BA]
5:     **if** AE[relations][execute_transformation_on] ∈SA entity **then**
6:       **if** SA[displaceable] == true **then**
7:        Continuous_SB ← SB
8:       **else**
9:        Non-Continuous_SB ← SB
10:       **end** **if**
11:     **else** **if** AE[relations][execute_transformation_on] ∈filtered_SA entity **then**
12:       **while** filtered_SA[relations][first_argument] ∉ SA entity **do**
13:         filtered_SA = filtered_SA [relations][first_argument]
14:       **end** **while**
15:       query SA entity from filtered_SA entity [relations][first_argument]
16:       **if** SA[displaceable] == true, **then**
17:         Continuous_SB ← SB
18:       **else**
19:         Non-Continuous_SB ← SB
20:       **end** **if**
21:     **end** **if**
22:   **end** **for**
23: **end** **procedure**

The scenarios that remove all BAs even if remove_at_every_loop is false are as follows: SBs are added or removed in real time, or geometric areas of non-displaceable SAs are changed in real time. An example of the latter is a real-time change of destination in Figure 2, which influences the lane that the robot needs to traverse. This clarifies line 2–6 of Algorithm 2, which generates and updates the behavior map from the (non)continuous SBs. Each loop removes the BAs that result from continuous SBs at lines 7–11. We, then, generate BAs from the continuous SBs or the not yet analyzed non-continuous SBs from line 13 onward. For each SB, we retrieve the AE entity at line 15 to retrieve the geometric areas. If the TSQ is applied on an SA entity, then line 17 executes it on all its geometric areas. If the entity is a filtered_SA entity at line 18, then we need to resolve the filtered_SA first by resolving the “deepest” filtered_SA entities that may be connected to the relations first_argument or second_argument at line 19. For each retrieved geometric area at line 22, we, then, create a BA data structure similar as the one mentioned above from the resolved geometric areas and the SB entity properties/relations at lines 23–35.

Algorithm 2 Procedure to generate and continuously update the behavior map.

1: **procedure** GenerateAndUpdateBehaviorMap
2:   **if** initialization program ∨ SBs are updated **then**
3:     - Analyzed_Non-Continuous_SBs = []
4:     - clear all BAs from behavior map
5:     - run Alg. 1 to store continuous and non-continuous SBs.
6:   **end** **if**
7:   **for** each BA∈ behavior map **do**
8:     **if** BA[remove_at_every_loop] = = true **then**
9:        - remove BA from behavior map
10:     **end** **if**
11:   **end** **for**
12:   **for** each SB **do**
13:     **if** SB ∈ Continuous_SB ∨ (SB ∈Non-Continuous_SB ∧ SB ∉Analyzed_Non-Continuous_SB) **then**
14:        - considered_geometric_areas = []
15:        - query AE entity from SB[relations]       [description_geometry_BA]
16:        **if** AE[relations][execute_transformations_on] ∈ SA entity **then**
17:          - considered_geometric_areas ← retrieved geometric areas of SA from semantic map
18:        **else** **if** AE[relations][execute_transformations_on] ∈ filtered_SA entity **then**
19:          - retrieve the geometric areas of the entities at relations first_argument and second_argument
20:          -considered_geometric_areas ←geometric areas from filtered_SA
21:        **end** **if**
22:        **for** each geometric area ∈ considered_geometric_areas **do**
23:          -create new BA = { }
24:          -create BA[coordinates_x] and BA [coordinates_y] from geometric area description
25:          **if** SB Continuous_SB **then**
26:            -BA[remove_at_every_loop] = true
27:          **else** **if** SB ∈ NonContinuous_SB **then**
28:            -BA[remove_at_every_loop] = false
29:          **end** **if**
30:          -retrieve EB entity from SB [relations][execute_EB]
31:          -BA[EB_type] = EB [properties][EB_type]
32:          BA[EB_parameter_type] = EB [properties][EB_parameter_type]
33:          BA[EB_parameter_value] = SB [properties][EB_parameter_value]
34:          BA[intention_type] = SB [properties][intention_type]
35:          -behavior map ← BA
36:       **end** **for**
37:     **end** **if**
38:   **end** **for**
39: **end** **procedure**

#### 4.2.2 Formulation of the constraint-based optimization problem

This paper advocates the use of COP-based “low-level controllers” because they represent a family of motion controllers that are, both, able to make optimal use of a robot’s physical capabilities and *numerically* configurable from *symbolic* behavior maps (Section 4.2.5). The generic form of a COP is as follows:

$$\begin{array}{cc} robot\;state\;\&\;domain: & \;q\in Q\\ input\;state\;\&\;domain: & \;u\in U\\ behavior\;map\;state\;\&\;domain: & \;b\in B\\ objective\;function: & \;\underset{u}{\min}f\left(q,u,b\right)\\ & \;=\sum_{k=1}^{\dim U}f_{k}\left(q,u,b\right)\\ equality\;constraints: & \;g\left(q,u,b\right)=0\\ inequality\;constraints: & \;h\left(q,u,b\right)\leq 0. \end{array}$$ (1)

The state $q={\left(\begin{array}{ccc} x & y & \theta \end{array}\right)}^{T}$ is the position and orientation of the robot. The input state $u={\left(\begin{array}{cc} v & \omega \end{array}\right)}^{T}$ contains the translational and rotational velocities, shown in Figure 6A. The behavior state *b* represents the spatial relation between the robot position and the BAs on the behavior map (e.g., its overlap with an *avoid* area), which is the source of the COP’s (in)equality constraints. A COP solver algorithm then computes the “optimal” input *u* that respects the (in)equality constraints and minimizes an objective function *f*, which is a summation of input-related objectives *f*_*k*_ (Section 4.2.5). The COP’s numerical values are configured by relating them to the EB_parameter_value in the BA data structure, in Algorithm 3.

Algorithm 3 Procedure to generate the COP from the BAs.

1: **procedure** GenerateCOP
2:     *//input for COP generator*
3:   - list_objectives = [], list_constraints = []
4:   - monitor retrieves relevant BAs from the behavior map
5:   - list_objectives, list_constraints ← control specifications from relevant BAs
6:     *//generate the COP from list of constraints and objectives*
7:   - deduce *f*_*k*_ and *f*_*k*, inf_ from ‘list_objectives’ and priority of intentions
8:   - list_constraints ← constraint replacement from *f*_*k*, inf_ if available
9:   - remove duplicate constraints and inferior intention constraints from ‘ list_constraints’ that refer to the same function_id
10:   - formulate the COP in Eq. 1
11: **end** **procedure**

#### 4.2.3 Monitors

**FIGURE 7:**
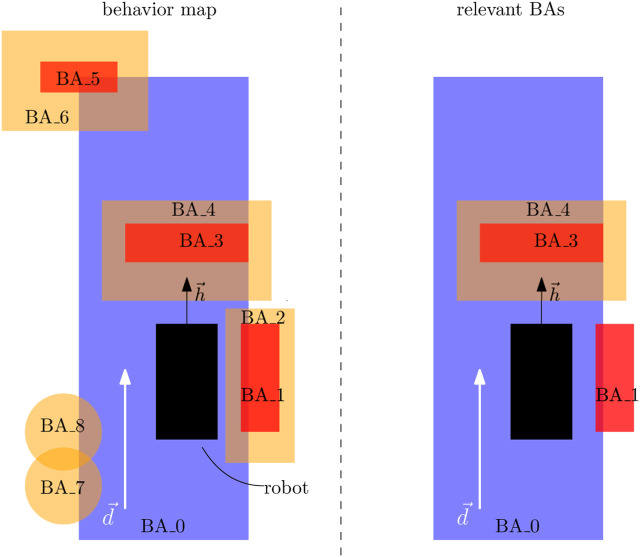
Example of a behavior map on the left with its relevant BAs on the right.

Each BA triggers the execution of an EB, which, in turn, generates a set of objectives and constraints. To reduce the amount of control specifications to consider in a COP, only the *relevant* BAs are taken into account (line 4, Algorithm 3). For example, BA_7 and BA_8 in Figure 7 (left) are too far behind the robot and BA_5 and BA_6 are too far in front of the robot. *Monitors* are the mechanism to decide about the relevance of BA types because they introduce limits on the search for the satisfaction of a FSQ. For example,

monitor_BA_drive = { FSQ: Intersects, first_argument: BA_drive, second_argument: SA_robot }

specifies that a drive area is only relevant if it intersects with the robot. The right of Figure 7 depicts an example of the relevant BAs after applying the monitor.

#### 4.2.4 EBs and control specifications

Each symbolic EB is translated into an EB_control data structure (Figure 5), such as

EB_drive_control = {
objectives: [AlignDirection, MaximizeTranslationalSpeed]
constraints: [SpeedLimitTrans, SpeedLimitRot]

From this, one can derive objectives of types “AlignDirection” and “MaximizeSpeed” and constraints of types “SpeedLimitTrans” and “’SpeedLimitRot.” Each BA advocates the execution of an EB_control and parameterizes the generated objectives and constraints at line 5 of Algorithm 3, which is also observed via the dashed arrows in Figure 5. The objective “AlignDirection” minimizes the difference between the robot heading $\vec{h}$ (Figure 6A) and a direction vector $\vec{d}$ (Figure 6B), and the objective “MaximizeTranslationSpeed” maximizes the velocity in the direction of $\vec{h}$ , that is, input *v*. The constraint “SpeedLimitTrans” restricts the translational velocity *v*, and the constraint “SpeedLimitRot” restricts the rotational velocity *ω*. The BA mentioned above could then generate the following control data structures:

objective_1 = {
function_id: AlignDirection,
parameter_type: [’direction vector’],
parameter_value: [[1,0]],
intention_type: ’Progress’,
constraint_replacement_fid: MaxAngleLimit,
constraint_replacement_par_type: [’angle_diff’],
constraint_replacement_par_value: [60],
input_type: rotational velocity
}
objective_2 = {
function_id: MaximizeTranslationalSpeed,
parameter_type: [],
parameter_value: [],
intention_type: ’Progress’,
constraint_replacement_fid: none,
constraint_replacement_par_type: [],
constraint_replacement_par_value: [],
input_type: translational velocity
}
constraint_1 = {
function_id: SpeedLimitTrans,
parameter_type: [’translational speed limit’],
parameter_value: [2],
intention_type: ’Progress’
}
constraint_2 = {
function_id: SpeedLimitRot,
parameter_type: [’rotational speed limit’],
parameter_value: [40],
intention_type: ’Progress’
}

Section 4.2.5 explains the members constraint_replacement_… and input_type of the objectives.

#### 4.2.5 Resolving objectives and constraints

Monitors filter BAs that are far away from the robot, but BAs can still overlap, as shown in Figure 7. A further reduction in relevant BAs comes from the *intention* semantic labels: the objectives and constraints with the highest priority *intention* are selected. This section assumes the following priority ranking for the examples: NoDamage > Safety > Progress.

To resolve a composition of constraints at line 9 of Algorithm 3, we remove the duplicates and from the constraints with similar function_id, we keep only those with the highest intention priority. For example, a speed limit constraint of 5 m/s can be introduced with an intention of progress and another similar speed limit constraint of 2 m/s can be introduced with an intention of safety. In this case, the highest intention priority is safety, resulting in dismissal of the 5 m/s speed limit. However, if progress would be temporarily more important, then the robot would dismiss the 2 m/s speed limit instead.

An additional mechanism is added to objective functions at lines 7–8. In contrast to *constraints* that can either be satisfied or not satisfied, *objectives* come with a scalar-valued “degree of satisfaction.” Whenever multiple objective functions are considered, each objective function could be multiplied by this scalar value to represent its relative importance. Typically, such weight values are manually tuned by the control engineers. Formal models that relate those weight values to the semantic map do not exist (yet), and, therefore, it is not intuitive for an application developer to do this tuning. It is, therefore, chosen to allow dismissals of objective functions and optionally replace them with constraints. The latter is added as one may not want to fully dismiss an objective function. For example, dismissing “Aligndirection” objective_1 mentioned above would introduce a constraint that would still guide the robot toward direction vector $\vec{d}$ but would restrict the angle difference between $\vec{h}$ and $\vec{d}$ to not exceed a limit. This clarifies the objective members constraint_replacement_… which give instructions on the replacement constraint and its parameterization. The last objective member input_type is added to only do this replacement procedure for objectives that will minimize the same kind of input type and are also of lower *intention* priority. For example, let us consider an additional objective:

objective_3 = {
function_id: AvoidArea,
parameter_type: [],
parameter_value: [],
intention_type: ’Safety’,
constraint_replacement_fid: none,
constraint_replacement_par_type: [],
constraint_replacement_par_value: [],
input_type: rotational velocity
}

In this case, objective_1 and objective_2 can both be satisfied in a decoupled manner, as objective_1 is mainly concerned about rotating the robot to align its heading, whereas objective_2 is concerned about pushing the robot as fast forward as possible. One can relate this to car driving, where objective_1 is related to rotating the steering wheel and objective_2 to pushing the gas pedal. The objective function description in Eq. 1 also clarifies this where the objective function *f* is a summation of input-specific objective functions *f*_*k*_ with objective_1 being *f*_1(=*ω*)_ and objective_2 *f*_0(=*v*)_. Objective_1 and objective_3, however, both cannot be satisfied in a decoupled manner as objective_3 also wants to rotate the robot to avoid areas, and there is, thus, ambiguity in the extent of how one should satisfy either of both constraints. For example, the left side of Figure 7 characterizes a situation where objective_1 wants the robot’s heading to remain unchanged, while objective_2 wants it to rotate anticlockwise. The *intention* knowledge in this case clarifies that objective_3 would be *f*_1_ with objective_1 being the lower-priority objective *f*_1, inf_ at line 7. The constraint replacement mechanism can then still satisfy the dismissed objective_1 to a certain extent by adding the constraint into the composition of constraints at line 8.

Executing Algorithm 3 to the relevant BAs in the right side of Figure 7 can result in the following list of objectives and constraints:

list_objectives = [objective_1, objective_2, objective_3]
objective_1 = {function_id: MaximizeSpeed, input_type: translational velocity, intention: Progress,
constraint_replacement_fid: none, ⋯} (BA_0)
objective_2 = {function_id: AlignDirection, input_type: rotational velocity, intention: Progress,
constraint_replacement_fid: MaxAngleDiff,
constraint_replacement_par_type: [‘angle_diff’],
constraint_replacement_par_value: [60], .} (BA_0)
objective_3 = {function_id: AvoidArea, input_type: rotational velocity, intention: Safety,
constraint_replacement_fid: none, ⋯} (BA_4)
list_constraints = [const1, const2, const3, const4, const5, const6]
const1 = {function_id: SpeedLimitTrans, parameter_type: [’translational speed limit’],
parameter_value: [5],intention_type: Progress} (BA_0)
const2 = {function_id: SpeedLimitRot, parameter_type: [’rotational speed limit’],
parameter_value: [1],intention_type: Progress} (BA_0)
const3 = {function_id: SpeedLimitTrans, parameter_type: [’translational speed limit’],
parameter_value: [2],intention_type: Safety} (BA_4)
const4 = {function_id: SpeedLimitRot, parameter_type: [’rotational speed limit’],
parameter_value: [0.5],intention_type: Safety} (BA_4)
const5 = function_id: NoEnterArea, intention_type: NoDamage (BA_3)
const6 = function_id: NoEnterArea, intention_type: NoDamage (BA_1)
const7 = {function_id: MaxAngleDiff, parameter_type: [’angle_diff’],
parameter_value: [60], intention_type: Progress} (objective_2)

From the list of objectives, we first deduce the most important input-specific objectives *f*_*k*_ and the dismissed objectives *f*_*k*, inf_. In this case, “Safety” is more important than “Progress”; thus, *f*_0_ = objective_1 and *f*_1_ = objective_3 with *f*_1, inf_ as objective_2 at line 7. For the dismissed objectives, it is checked if replacement constraints are specified at line 8, which, in this case, would add const7 to the list of constraints. Line 9 removes duplicate constraints, that is, either one of const5 or const6, and the lower-priority *intention* constraints that refer to the same “function_id,” that is, const1 and const2. The COP can then be formulated from the filtered list of objectives = [objective_1, objective_3] and list of constraints = [const3, const4, const5, const7] at line 10.

So, finally, the sequence of algorithms Algorithm 1, Algorithm 2, and Algorithm 3 at every program loop generates a COP from the semantic map in real time.

## 5 Experimental validation

The contribution of this section is the practical validation of the applicability of the methodology in simulated and real-world environments, that is, whether it is, indeed, possible to transfer the complexity of the behavior design to the application layer with SBs, while keeping the design of the lower interaction and control layer more generic with, e.g., EBs. Settings are explained of the experiments in which we show behavior change by 1) changing the priority of intentions, 2) adjusting the behavior map, and 3) adjusting SBs with the real-time influence of perception and localization on the semantic map. Real-time environmental disturbances adjust the semantic map, which, in turn, adjusts the behavior map that generates the COP. Verification of the experiments is carried out by observing the behavior map and determining whether the robot executes the control instructions on it properly.

### 5.1 Experimental setting

**FIGURE 8:**
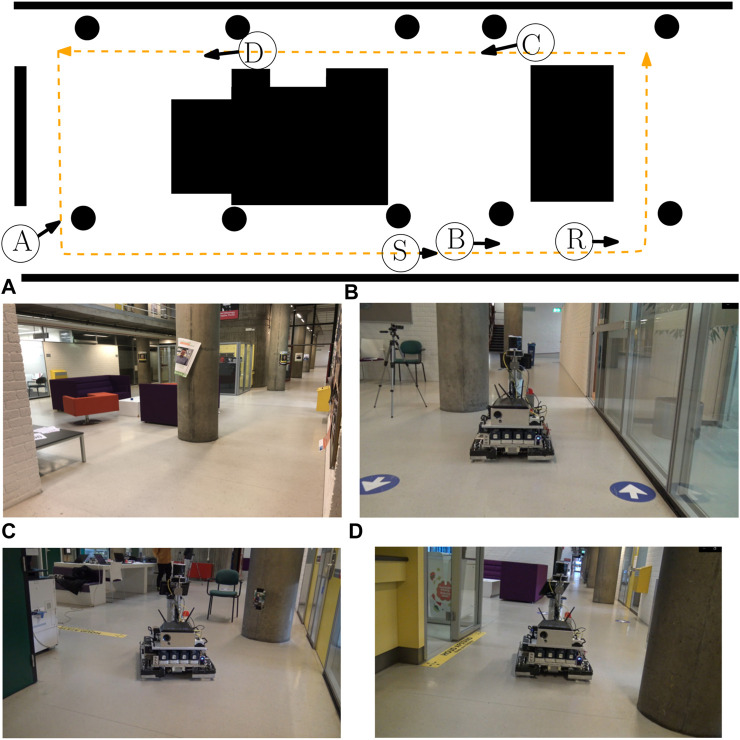
2D sketch of the experimental environment with the black areas denoting areas occupied by structural elements. The real-world environment is visualized by reporting images associated with the view-points A, B, C, and D. The orange dashed line represents the path that the robot took in the experiments for which the circle S represents the starting point of the robot in the simulations and the circle R represents the starting point of the robot in the real-world experiment.

**FIGURE 9:**
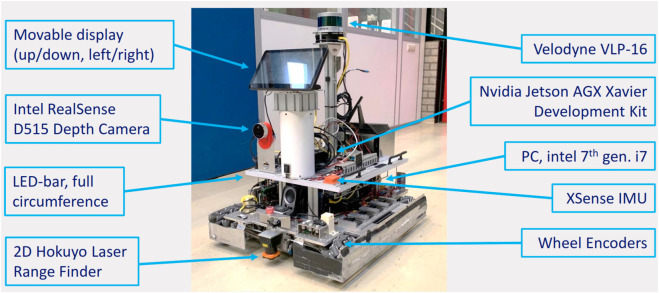
Frontal view of the mobile platform with hardware components indicated with blue arrows.

The indoor environment of Figure 8 is considered for the simulated and real-world experiments. The robot in Figure 9 is deployed with the following hardware characteristics: *omniwheels* for instantaneous translational/rotational motion, *Hokuyo UTM-30LX 2D Laser Range Finder (LRF)* for detecting the occupied areas, and *Intel Realsense D515 Lidar camera* for RGB images. The used software components are *Robot Operating System (ROS) Kinetic/Melodic* ^2^ for the communication within the robot, *YOLO* ^3^ algorithm for the detection of humans, *relational database PostgreSQL* ^4^ *with PostGis extension* ^5^ for storing and retrieving the semantic map and behavior map (geometric) properties, Python 2.7^6^ for the program execution, and *localization algorithm of the work of Hendrikx et al. (2021)* to store the robot position in the geometric and semantic maps.

**FIGURE 10:**
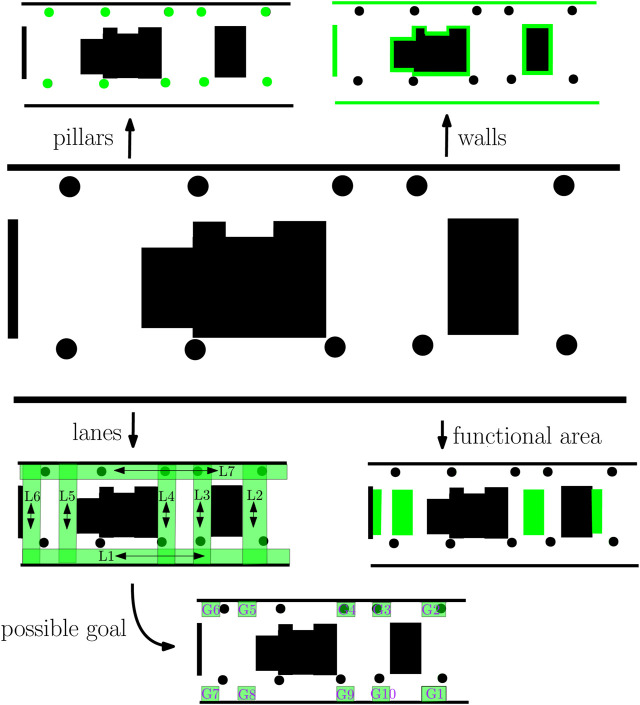
Visualization of the semantic labels and their geometric properties marked in green in the semantic map. At the left top, the green areas represent pillars, at the right top the walls, at the left bottom the lanes, at the bottom the goals, and at the right bottom the functional areas.

Figure 10 visualizes the SAs of the semantic map which where manually created and inserted into the database. At the moment, no approach exists that can generate such a semantic map. To increase the applicability, generic SAs were considered as 1) solid objects such as *walls* and *pillars*, 2) *lanes* serving as the area for the robot to navigate over, 3) a *goal* serving as the area for the robot to go toward, and 4) *functional areas* indicating areas that are used for other purposes than navigation, e.g., an area where people can sit to rest or talk such as the purple couches at the left bottom of Figure 8. In our semantic map, there is only one SA goal present, of which the geometric area is one of the possibilities indicated on the bottom of Figure 8. To increase the applicability of the methodology, it is necessary to reach an agreement in robotic community of the types of SAs that can be used. Similar collaboration can be set up as the design of knowledge schemas for linked data on the web^7^. Without such standardization, one can limit the design of SBs to generic abstract SAs as *Walls* instead of more detailed variations as *WoodenWalls*. The WoodenWall can be defined as a subclass of Wall and inherit the SB, where inheritance is a common technique in knowledge engineering (Tenorth and Beetz, 2017). The current methodology also promotes the usage of generic SAs, where FSQs allow the application developer to filter the SA instead of defining combinatorial SAs as “LaneThatContainsWallsAndRobot.”

To show a moving robot, a custom COP solver was designed to solve the generated COP from the robot program in Section 4. We kept the design of the individual components, such as the monitor and objectives/constraints, simple as it would have taken considerable time to guarantee quantitative criteria as robustness or time/distance optimality for them. Similar reasoning was made for the COP solver, as the design of an advanced COP controller could be a paper on itself (Mercy et al., 2018). Therefore, the focus of this work is solely on showing applicability of the methodology that it is, indeed, possible to transfer the complexity in behavior design to the application layer in the form of SBs, while keeping the layers “below” relatively generic with, e.g., EBs. Quantitative results are more related to an optimization of the lower control layer, for which an optimal design was not the focus of this work. Supplementary material is provided of the implementation details for the interested readers.

For the following experiments, one or more SBs of Table 1 was used. Simple SBs were designed to realize experiments, where a theorem of designing proper SBs is left for future work. This is reasonable as the traffic system even has a whole manual about this topic (DoT, 2009). It is explicitly specified in the table of which SBs are continuous and a description is given of the AE and BE. The continuous SBs allow the robot to react to unforeseen circumstances as these will update the behavior map real-time via Algorithm 2 at lines 7–10. Supplementary material is provided of a more detailed description of those SBs for the interested readers.

**TABLE 1:** Description of the designed SBs where the first column gives a short description of the SB, the second column shows whether it is continuous, the third column shows a short description of the AE and BE, and the fourth column shows the intention. Unclassified laserpoints are the laserpoints that are not contained within a no-enter area on the behavior map.

Description	Continuous SB	AE + BE	Intention
1. Stop at goal	No	Stop behavior on goal	CompleteTask
2. Drive on lane to goal	No	Drive behavior on lane that contains goal and robot	Progress
3. Avoid walls on lane to goal	No	Avoid area on wall that intersects with lane	Safety
4. Avoid pillars on lane to goal	No	Avoid area on pillar that intersects with lane	Safety
5. Avoid functional area (FA) on lane to goal	No	Avoid area on FA that intersects with lane	Safety
6. Avoid driving into pillars on lane	No	Avoid area in “front” of pillars	Safety
7. Avoid driving into FA on lane	No	Avoid area in “front” of FA	Safety
8. Stay in lane to goal	No	Avoid area around lane	Progress
9. No collision walls in lane	No	No-enter area on walls	NoDamage
10. No collision pillars in lane	No	No-enter area on pillars	NoDamage
11. Pass humans respecting comfort distance	Yes	Avoid area on human with shape HumanInDirection toward robot	Safety
12. No collision human in lane	Yes	Avoid area on human with shape HumanInDirection toward robot	NoDamage
13. No collision unknown laserpoints	Yes	No enter on unclassified laserpoint	NoDamage

### 5.2 Results

#### 5.2.1 Changing behavior via priority between intentions

**FIGURE 11:**
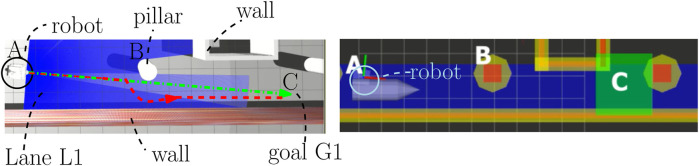
Simulation results of changing the order of intentions. On the left is the Gazebo environment (http://gazebosim.org/), and on the right is the behavior map in RVIZ (http://wiki.ros.org/rviz). A mobile robot will move from A to C while passing a pillar located at B. The green path is the executed robot motion by solving the COP real-time for intention “Progress” > “Safety,” and the red path is the executed robot motion by solving the COP for intention “Safety” > “Progress.”

Figure 11 displays the results of a simulation^8^ in which we change the motion behavior of the robot by only changing the priority between intentions, which influences the generated COP from Algorithm 3. The considered SBs are 1–5 and 8–10, and the SAs are shown in Figure 11 (left). We have visualized the results of the executed robot motion, by real-time solving of the COP, as two colored paths shown in Figure 11(left). It should be noted that these paths are not the result of offline path planning algorithms as our approach does not try to follow a predetermined path. The green path took 16 s and is the result of solving a COP with a priority “Progress” > “Safety,” where the red path took 24 s with a priority “Safety” > “Progress.” This result is not surprising, as being safer is realized by driving slower and/or precautiously moving away from objects.

We verified a correct execution of the methodology by observing the robot in the behavior map as in Figure 11 (right) with the designed monitors and SB configurations in mind. The no-enter behavior is checked by verifying that the robot will never overlap with these red areas. The drive behavior is verified by checking whether the robot drives aligned to the direction array $\vec{d}$ (toward the right). The avoid behavior is verified by checking whether the robot rotates away from those orange areas and slows down. The stop behavior is verified by checking whether the robot stops its motion whenever it is contained in the green area. Indeed, this is the case, where the main difference between the two paths is the avoid area at point B, resulting from SB 4. Whenever “Safety” is more important, this area would be avoided by the robot while simultaneously lowering its speed. In contrast to when “Progress” is more important, we, indeed, verify that the robot ignores these avoid areas.

#### 5.2.2 Changing behavior real time by adding and composing behavior areas

**FIGURE 12:**
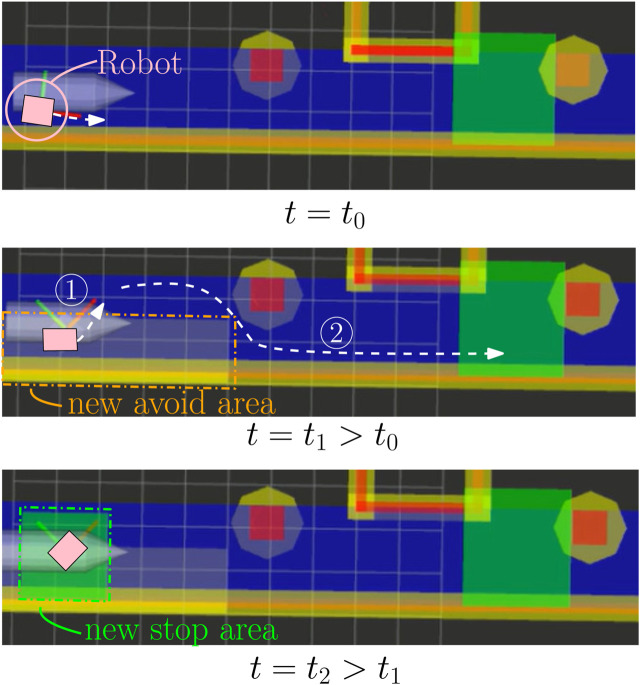
Simulation results of adding and composing BAs with the behavior map. The mobile robot (pink square) will move right toward the green stop area. The executed robot motion is visualized as the white dashed arrows. At time *t* = *t*_1_, a permanent avoid area is added on the robot, and at time *t* = *t*_2_, a temporary stop area is added on the robot.

Figure 12 displays the execution motion results in a simulation environment^9^ in which we influenced the robot behavior by adding independently new BAs in real time. We execute the SBs 1–5 and 8–10 where the visualization of SB 8 is missing. We add a permanent avoid area from time *t* = *t*_1_ and a temporary stop area at *t* = *t*_2_ which lasts for 4 s, as data structures.

BA_1 = {
EB_type: avoid
EB_parameter_type: [’translational speed limit’, ’rotational speed limit’],
EB_parameter_value: [0.2 m/s, 0.35 rad/s],
intention_type: ’Safety’,
coordinates_x: [⋯],
coordinates_y: [⋯]
remove_at_every_loop: false
},
BA_2 = {
EB_type: stop
EB_parameter_type: [],
EB_parameter_value: [],
intention_type: ’NoDamage’,
coordinates_x: [⋯],
coordinates_y: [⋯]
remove_at_every_loop: false
}.

These BAs could have resulted from some SBs as “avoid water spillage area” and “stop for passing human.” Also, the methodology is structured enough to handle the composition of BAs such that an application developer can design SBs without the consideration of the already present SBs. Subsequently, we do not need to check for consistency or event transitions between behaviors. We, however, cannot guarantee that a non-zero input will be found, that is, the robot will not move. When more and more BAs are added, it can occur that a composition of constraints is obtained that can only be solved with a zero input. Also, similar to the traffic system, a manual can be defined (DoT, 2009) containing the best practices of adding BAs, but this is out of scope in the current work.

We verify the correct execution of robot motion via the behavior map. At the robot’s initial configuration at time *t*_0_, we would expect the robot to take the avoid area into account and turn slightly anticlockwise while driving toward the green stop area. This, indeed, occurs as indicated by the dashed white arrow on top of Figure 12. At time *t*_1_, the custom avoid area is added such that we expect the robot to slow down and rotate further anticlockwise toward the blue area that has no overlap. This is, indeed, the case as visualized by the white arrow numbered 1 in the middle of Figure 12. At time *t*_2_, a stop area is added on top of the robot and we expect the robot to stop, which, indeed, occurs at the bottom of Figure 12 where no white arrow is present. After 4 s, the stop area is removed and we expect the robot to squeeze itself in the non-overlapping blue area between the pillar and the custom avoid area. It should be noted that SB8 makes an avoid area around the drive lane which is not visualized. Indeed, the robot behaved as expected where its resulting motion is indicated in the middle of Figure 12 with the white arrow numbered 2.

#### 5.2.3 Reconfiguring the behavior map

**FIGURE 13:**
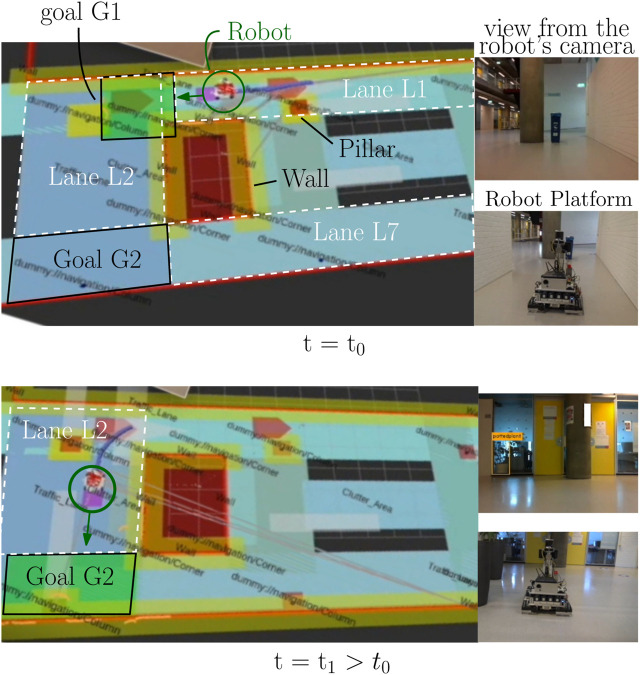
Real-world experimental results of real-time behavior map changes by changing the semantic map. The relevant goal is represented as a green stop area, the robot is encircled in dark green, and the lane is indicated as a white-dashed polygon. At time *t*_0_, the relevant goal for the “‘drive to goal” SB is G1 on the semantic map, and subsequently, a green stop area is placed there. At time *t*_1_, the relevant goal changes to G2, and subsequently, the green stop area changes its position.

Figure 13 displays the results of the real-world experiments^10^ in which we show the applicability of the methodology in combination with the localization algorithm of the work of Hendrikx et al. (2021). This localization algorithm will update the robot its position in the semantic and behavior maps. All the SBs in Table 1 are active in this experiment. An application developer updates the geometric area of the goal from G1 to G2 whenever the robot has reached G1, where a goal is considered as a non-displaceable SA. This invokes a recalculation of the behavior map as in Algorithm 2 lines 2–5. We notice this in Figure 13 as the bottom part has 1) a different green stop area and 2) additional orange avoid areas at the bottom of G2 due to the wall that intersect with lane L2. It should be noted that RVIZ did not always properly remove the BA visualization, but via the observed robot motion behavior, we did conclude that BAs were removed.

At time *t*_0_, the geometric area of the goal is equal to G1, and according to SB1, a green stop area needs to be put on top of it. The lane that contains this goal and also intersects with the robot is L1, and hence, a blue drive area is put on lane L1. At time *t*_1_, as in the bottom of Figure 13, the robot has passed goal G1 and the application developer reassigns the geometric area of the goal to become G2. Updated SB1 now puts the green stop area on goal G2, and the updated SB2 adds a blue drive area on top of lane L2 with a direction vector toward G2. It is, therefore, that the robot now moves “downward.”

#### 5.2.4 Dealing with dynamic or unknown objects

**FIGURE 14:**
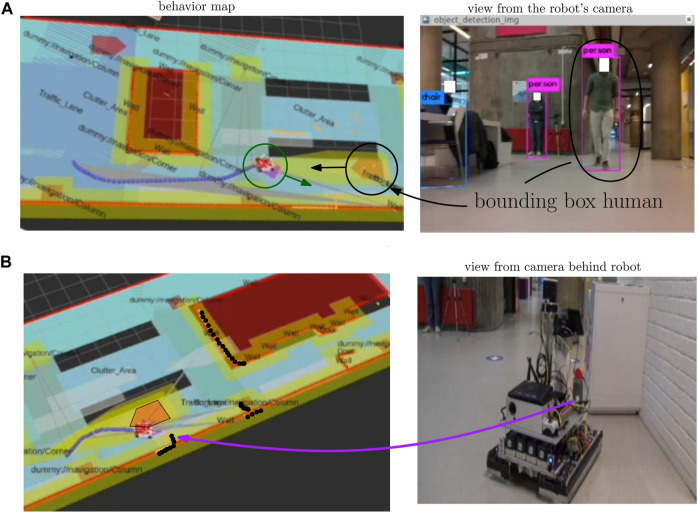
Real-world experimental result of the influence of the perception on the behavior map. The localization component adds the geometric and semantic robot property onto the semantic map (dark green circle), whereas the perception component adds the detected human and unclassified laserpoints on the semantic map. The SBs then generate the proper behavior map from this updated semantic map.

Figure 14 displays the influence of the perception component on the behavior map, where the robot has passed goal G2 and is on its way to goal G6 of Figure 10 by traversing lane L7. An unforeseen circumstance occurs where there is a human moving toward the robot at Figure 14A. On the right, the image output of the intel real sense is seen, where two humans are detected via the two bounding boxes. Only the black encircled human will influence the behavior map as the other is not present on lane L7. This human then occurs on the behavior map according to the shape of the HumanInDirection SQ in Figure 4. The perception component then influences the behavior map by adding the human object onto the semantic map, which gets translated by SB11 and SB12 into the behavior map.

In Figure 14B, a situation is created where the human has just left the robot’s vision and the robot is in front of a trashbin not priorly known. The BAs from SB11 and SB12 are still active where we emphasized the no-enter area of the human on the left. The black dots on the behavior map represent the laserscan points, and currently, most are contained within the no-enter areas of the behavior map. The part that belongs to the trashbin (indicated with the purple arrow) is not contained within a priorly known no-enter area. This is because 1) we were not aware of its existence and, thus, have not added it *a priori* on the semantic map and 2) no SB was designed that would do something with trashbin detections. In this case, the laserpoints of the trashbin are deemed “unclassified” and we add them as no-enter areas on the behavior map. The no-enter area of the trashbin and the human makes it sure that the robot cannot move further. However, after the human BA is removed, the robot can pass the trashbin from the left side.

## 6 Conclusion

### 6.1 Discussion

The *technical* contribution of this paper is a robot programming methodology to adapt the low-level motion control specifications to the spatial context by means of a semantically annotated map. However, the biggest impact is expected from how the *three-layered structure* of the technical contribution distributes the *responsibilities of the programming* of mobile robot applications between the *control engineers* at the robot vendor’s side and the *application developers* at the robot user’s side. The latter “program” their application by adapting semantic areas on a map *to influence* (but not *to command*) the robots’ motion actions. To do so, they have a set of *semantic behavior* primitives at their disposal, and our desire is to have such a set (eventually, sooner, or later) *standardized* by a consortium of robot vendors. The control engineers in each such vendor are responsible for letting their robots interpret the semantic behavior map and turn them in efficiently executed robot motion. The overall *state of the practice* in the domain of mobile robotics can then evolve gradually and incrementally with new versions of the standardized semantic primitives. Not unlike the approach that is behind the tremendous success of “the Web” over the last 30 years, driven by evolving and vendor-neutral standardization of the HTML, CSS, and Javascript primitives. The focus of this paper is on the semantic behaviors, and the relevant “Key Performance Indicators (KPIs)” are the semantic *consistency* and *composability*: to what extent can application developers be sure that their combinations of semantic behaviors in geometric areas on a map result in the expected behavior of the robots? The current state of our research cannot yet *measure* these KPIs in a quantitative way, so our contribution is *to explain* how the presented set of semantic primitives and its underlying three-layer approach are *designed* with consistency and composability in mind, in such a way that scientific refutability can be applied to the various individual design decisions that we have made.

In real-world robotics applications, a major KPI is “performance”: how quickly, accurately, and reliably will the generated robot motions be? This design driver is (only) indirectly in scope of this paper: we advocate using a constraint-based optimization approach (such as *model-predictive control*) for the low-level motion control. This approach is not yet popular in the mobile robotics industry, not in the least, because it is significantly more complex than the mainstream approach of instantaneous velocity-based trajectory. However, its higher complexity is exactly of the type that is needed to exploit the semantic richness, and the online reactivity, that comes from the contributions of this paper. The “optimization” complexity is, almost by definition, also the enabler of higher performance. It is, however, too soon to be able to provide meaningful system-level performance measures.

From a technical point of view, the transformation from the semantic layer to the control layer (that is an *essential* component of the presented approach) is more complex than “just” filling in motion control templates (as they exist in some higher programming languages such as C++ or Java) with numerical parameters that are derived from the symbolic representations of semantic behaviors. Indeed, there exist many dependencies between these numerical parameters, such as priorities, or other non-linear constraint relations. Hence, techniques from formal knowledge representation and reasoning are required. Also, the success of the “semantic Web” technologies (RDF, OWL, JSON-LD, … ) is an excellent inspiration for our future work. In the current implementations behind the experiments reported in this paper, we have experimented with graph databases (such as Gremlin and Grakn) and with RDF/JSON-LD tooling (such as the Python rdflibs library), but eventually, we had to go to an *ad hoc* implementation of the reasoning, mostly because of the low maturity of the “real-time code generation” that is currently available in the mentioned “knowledge-based” toolchains.

This paper introduces a *methodology* to compose behavior areas which guide the robot’s motion, but it still lacks complete and constructive *guidelines* about *how* to choose and design behavior areas for a given application context, i.e., how to design proper semantic behaviors. The paper also does not guarantee that the methodology yields a geometric map with behavior areas that are consistent, optimal, or even free of decision-making conflicts. We do hypothesize that the methodology is a step forward toward supporting 1) application developers to create more complex systems, via only the semantic configuration of geometric maps and in a way that avoids vendor lock-in and 2) robot vendors to create semantically richer “interface” standards that can lift the level of applications in the whole domain and not just from one single vendor. Our research hypothesis is inspired and corroborated by a primary successful example in the (non-robotic) real world, namely, the traffic system, where the traffic layout consists of “behavior areas,” and the traffic code provides the semantic relations (priorities, objective functions, … ) that *allow* (but do not guarantee in themselves) safe and effective driving behavior under all circumstances and in all situations.

### 6.2 Future work

#### 6.2.1 Composability of elementary behaviors

The authors are not aware of a formal mechanism that can guarantee the creation of a *proper* geometric map with BAs, that is, the one from which a COP can be passed to the low-level motion controller, with a guarantee that conflicting motion constraints are avoided. In our current implementation, the COP is realized via decision trees where we 1) first retrieve the relevant EBs via the robot monitors and 2) resolve the compositions of EBs via priority rules based on the intentions of which the EBs are placed. In the situation when more EBs or types of intention are introduced, such decision trees become more difficult to design manually and manage.

One way to facilitate the creation of such decision trees is introducing more relations that interlink the intentions. For example, one could implement a hierarchy system on the intentions such that one can enact BAs in the same manner as long as they contain subintentions of the same superintention. For such more complex reasoning procedures, one may benefit by using a knowledge base such as the work of Waibel et al. (2011), Tenorth and Beetz (2017), and Beetz et al. (2018). Another way to facilitate the creation of such decision trees is using some automatization tooling. Almost certainly, some form of supervisory controller synthesis methodology, such as that in the work of Kok et al. (2021), will play a central role in tooling to create conflict-free composition of EBs.

#### 6.2.2 Perception in behavior areas

The BAs, as of now, only suggest behavior related to the *motion* capabilities of the robot. We, however, do foresee possibilities of extending the BAs to include instructions about the robot’s *perception* capabilities. This is also present in the traffic system, where warning signs such as “you are nearing a dangerous intersection” are present to raise the awareness of the drive (DoT, 2009). The BAs could then either suggest 1) to use certain kind of sensors or 2) to indicate the direction of interest to where the sensors should look at. For example, if there is no wall present in the map on the left of the robot, the robot controller would not need to execute sensor processing algorithms to detect walls in that area.

#### 6.2.3 Multi-robot interaction and coordination

In this work, the semantic behaviors are designed with a single mobile robot in mind. Of course, multiple robots can read from the same semantic map and realize coordinated movement via the proper generation of the behavior map via SBs. Improvements can then be made for the behavior map by 1) introducing new formal relations that represent multi-robot coordination dependencies, e.g., inspired by existing real-world traffic rules, and 2) allowing individual robots to share information in the form of SAs via a common geometric map, e.g., information of perceived obstacles.

## Data Availability

The original contributions presented in the study are included in the article/Supplementary Material. Further inquiries can be directed to the corresponding author.

## Footnotes

1
https://www.json.org/json-en.html

2
http://wiki.ros.org/kinetic, http://wiki.ros.org/melodic

3
https://pjreddie.com/darknet/

4
https://www.postgresql.org/

5
https://postgis.net/

6
https://www.python.org/download/releases/2.7/

7
https://schema.org/

8 video_1 for the interested readers.

9 video_2 for the interested readers.

10 video_3 for the interested readers.
