# Supplementary material for: Behavior adaptation for mobile robots via semantic map compositions of constraint-based controllers
Source: Front Robot AI. 2023 Aug 17;10:917637. doi: 10.3389/frobt.2023.917637 (PMC10470113; doi:10.3389/frobt.2023.917637)
Supplement: Supplementary file 2 [file DataSheet1.pdf]

Supplementary Material

1 REALIZATION OF THE ROBOT PROGRAM

1.1 Objectives and constraints

Table S1 contains the designed objectives and constraints. The first three rows represent objective functions and the other rows constraints. The objective AlignDirection minimizes the angle difference between the robot heading $\vec{h}$ and a direction vector $\vec{d}$ as seen on the left of Fig. S1. The variable $\vec{h}(\theta + \omega \cdot t)$ represents a future robot heading after applying a suggested input ω for a duration of t seconds. Here the t is related to a single iteration of the program. The objective MaximizeTranslationalSpeed maximizes the velocity v. The objective AvoidArea minimizes the overlap between an avoid area and a predicted robot footprint on which an input v and ω is applied, that is, SA_robot_input. This is visualized in the second column of Fig. S1 and this makes sure that the robot moves away from avoid areas. The calculations are done via a postgis query. The two speed limit constraints constrain the input of the robot as visualized in the third column of Fig. S1. The MaxAngleDiff constraint restricts the difference of angle between the heading $\vec{h}$ and $\vec{d}$ to not exceed a limit as seen in the fourth column of Fig. S1. The NoEnterArea constraint makes sure that the predicted robot footprint will never intersect with a no-enter area.

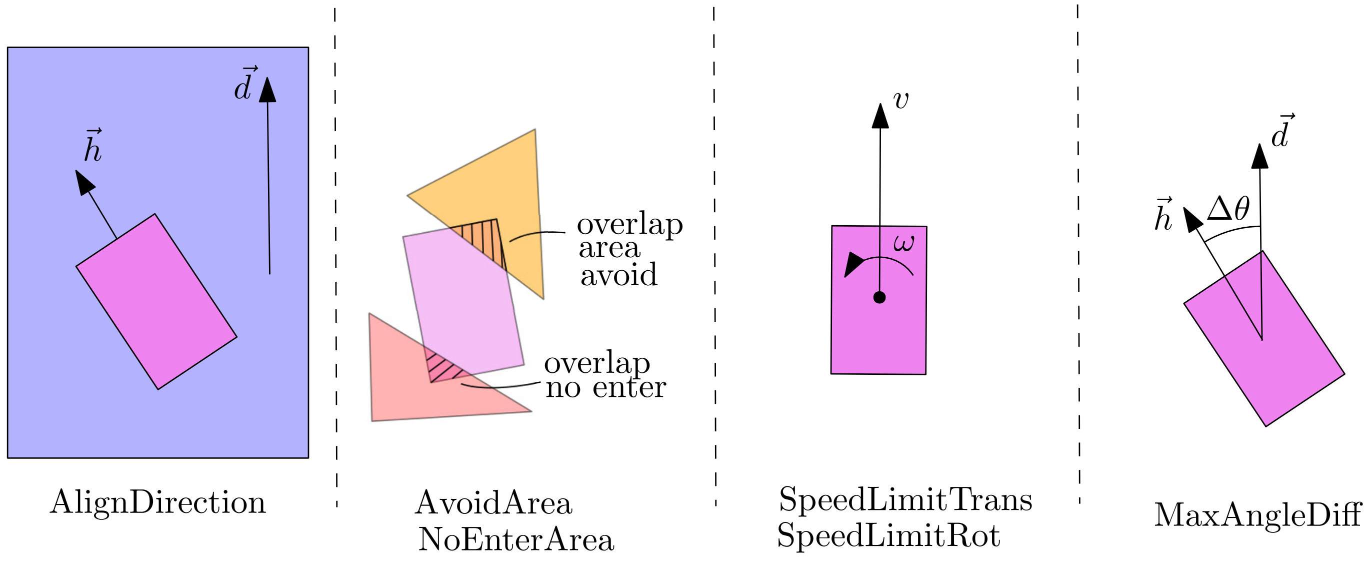

Figure S1. Additional visualizations to aid in understanding the calculations involved in the designed objectives and constraints. The related objectives and constraints are explicitly mentioned below the figure.

Table S1. Descriptions of the designed objective functions and (in)equality constraints. The first column describes the function id, the second column the input type, the third column any parameters that can be influenced by the application developer, the fourth column any replacement constraints with in brackets the parameterization, and the fifth column the equation or Spatial Query that is checked.

function_id	input_type	parameter	replacement	equation/SQ
AlignDirection	ω	$\vec{d}$	MaxAngle Diff (θ_{limit} = 60)	$\min_{\omega}(\arccos(\frac{\vec{h}(\theta+\omega\cdot t)}{\lvert\vec{h}(\theta+\omega\cdot t)\rvert}\cdot\frac{\vec{d}}{\lvert\vec{d}\rvert}))$
Maximize Translational Speed	v	N.A.	N.A.	$\min_{v} -v$
AvoidArea	ω	N.A.	N.A. N.A.	$\min_{\omega}$ Area(Intersects(SA_Robot_Input, BA_avoid))
SpeedLimit Trans	N.A.	v_{limit}	N.A.	v-$v_{limit} \leq 0$
SpeedLimit Rot	N.A.	ω_{limit}	N.A.	ω-$\omega_{limit} \leq 0$
NoSpeedTrans	N.A.	N.A.	N.A.	$v = 0$
NoSpeedRot	N.A.	N.A.	N.A.	$\omega = 0$
MaxAngle Diff	N.A.	θ_{limit}	N.A.	$\arccos(\frac{\vec{h}(\theta+\omega\cdot t)}{\lvert\vec{h}(\theta+\omega\cdot t)\rvert}\cdot\frac{\vec{d}}{\lvert\vec{d}\rvert})) - \theta_{limit} \leq 0$
NoEnter Area	N.A.	N.A.	N.A.	Intersects(SA_Robot_Input, BA_NoEnter) = False

1.2 Elementary Behavior and control specifications

The designed control specifications of the EBs are given as

```
EB_stop_control = {
    objectives: [],
    constraints: [NoSpeedTrans, NoSpeedRot]
},
EB_drive_control = {
    objectives: [AlignDirection, MaximizeTranslationalSpeed],
    constraints: [SpeedLimitTrans, SpeedLimitRot]
},
EB_avoid_control = {
objectives: [AvoidArea],
constraints: [SpeedLimitTrans, SpeedLimitRot]
},
EB_noenter_control = {
    objectives: [],
    constraints: [NoEnterArea]
}
```

The stop control is designed to stop all control inputs of the robot. The drive control is inspired from a traffic lane where a direction needs to be followed with speed limits. The avoid control wants to evade avoid areas where the speed limits are added to reduce the speed as these avoid areas are typically created as a precautionary measure for collision. The no-enter control does not permit the robot to ever enter a no-enter area.

1.3 COP solver

Algorithm 1 explains the procedure to obtain the optimal input $\vec{u}$. We will clarify this procedure via Fig. S2 in which a robot is depicted with its five predicted positions after executing discretized inputs $u1$ = (v=0.1, ω=40), $u2$ = (v=0.1, ω=20), $u3$ = (v=0.1, ω=0), $u4$ = (v=0.1, ω=-20), $u5$ = (v=0.1, ω=-40). We will assume that the BAs generate a COP with objectives and constraints:

```
objective_1 = {
    function_id: MaximizeSpeed,
    parameter_type: [],
    parameter_value: [],
    intention_type: Progress,
    constraint_replacement_fid: none,
    constraint_replacement_par_type: [],
    constraint_replacement_par_value: [],
    input_type: translational velocity
},
objective_2 = {
    function_id: AvoidArea,
    parameter_type: [],
    parameter_value: [],
    intention_type: Safety,
    constraint_replacement_fid: none,
    constraint_replacement_par_type: [],
    constraint_replacement_par_value: [],
    input_type: rotational velocity
},
constraint_1 = {
    function_id: SpeedLimitTrans,
    parameter_type: ['translational speed limit'],
    parameter_value: [0.1],
    intention_type: Safety
}
constraint_2 = {
    function_id: SpeedLimitRot,
    parameter_type: ['rotational speed limit'],
    parameter_value: [40],
    intention_type: Safety}
constraint_3 = {
    function_id: NoEnterArea,
    parameter_type: [],
    parameter_value: [],
    intention_type: NoDamage}
constraint_4 = {
    function_id: MaxAngleDiff,
    parameter_type: ['angle_diff'],
    parameter_value: [60],
    intention_type: Progress
}
```

At line 4 it is checked whether an input u can be found that satisfies all constraints. Immediately it can be observed that inputs $u2$ - $u5$ violate `constraint_3`. Input $u1$ does not violate `constraint_3` but does violate `constraint_4`, such that none of our inputs are valid. We then continue at line 5-6 and check whether all our remaining constraints are of the highest intention. Considering we rank intention as NoDamage>Safety>Progress, then we can deem that not all our remaining constraint are of intention NoDamage. We then continue at line 9 and temporarily dismiss the constraints belonging to the most inferior intention Progress, that is, `constraint_4`. 'Temporarily' implies only to be valid for the current program loop, where this dismissal is forgotten at the next program loop. We then start again at line 4 and notice that input $u1$ is now valid and execute it. This constraint dismissal mechanism is added for the situation in Fig. S2 and Fig. S3. The latter is a lane changing mechanism which frequently occurs in the real world experiments at Sec. 5.2.3 in the main text. Whenever a robot transitions from lanes as from Fig. S3(A) to Fig. S3(B), it will often have a heading configuration that violates the MaxAngleDiff constraint. By dismissing it temporarily, we allow it to turn its heading towards the direction vector of the new lane.

1.4 Monitor

We have designed the following monitors which check the satisfaction of an FSQ as

```
monitor_BA_stop = { FSQ: Contains, first_argument: BA_stop, second_argument: SA_robot }
monitor_BA_drive = { FSQ: Intersects, first_argument: BA_drive, second_argument: SA_robot }
monitor_BA_avoid_1 = { FSQ: Intersects, first_argument: BA_avoid, second_argument: SA_robot }
monitor_BA_avoid_2 = { FSQ: Intersects, first_argument: BA_avoid, second_argument: SA_robot_forward}
monitor_BA_noenter = { FSQ: Intersects, first_argument: BA_noenter, second_argument: SA_robot_around
}
```

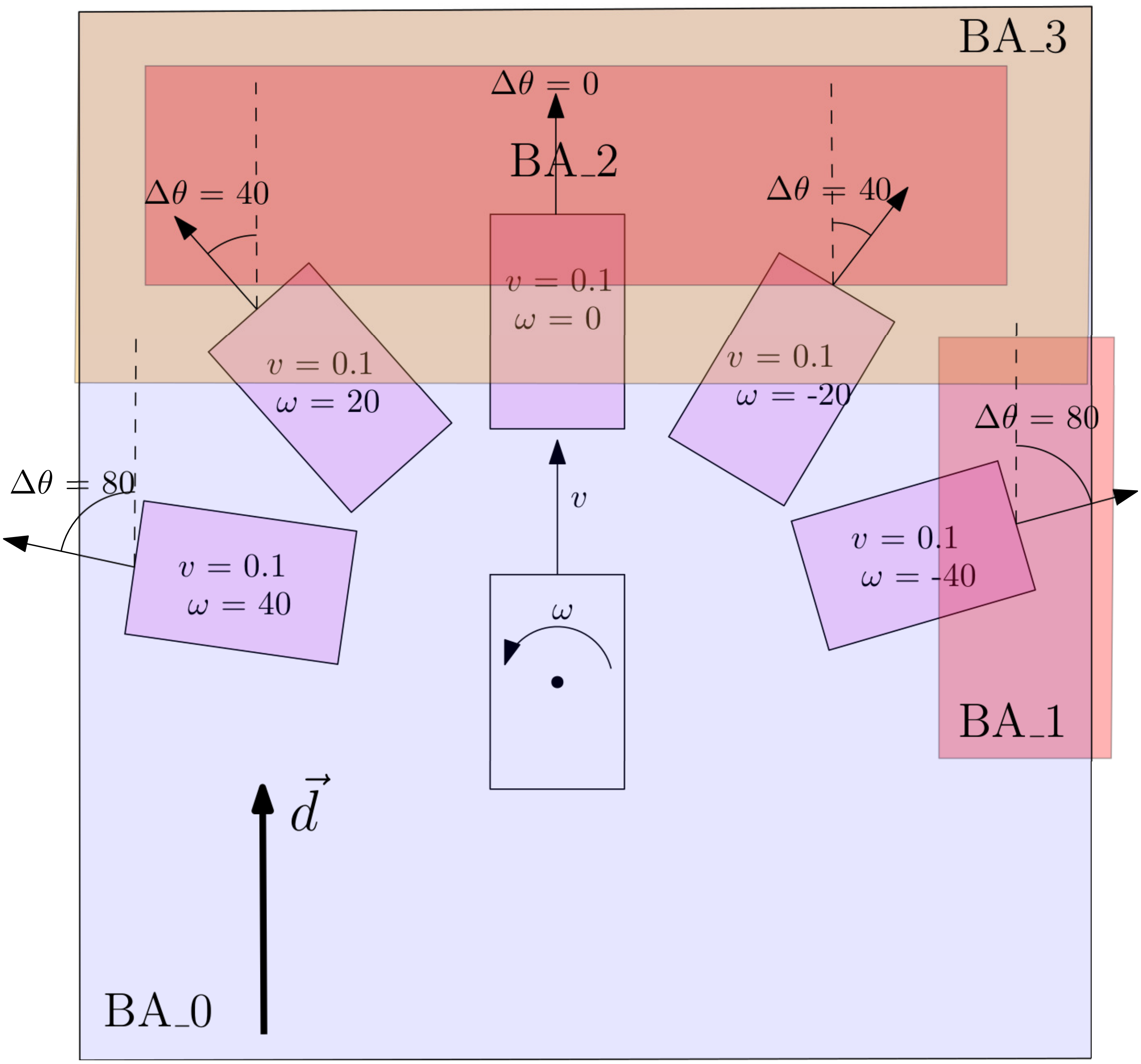

Figure S2. A mobile robot is depicted in the middle where its predicted footprints (pink squares) are visualized after applying the suggested control input. Four Behavior Areas are present: BA_0 is a drive forward area, BA_1 and BA_2 a no enter area, and BA_3 an avoid area.

A stop area is only relevant when the current geometric area of the robot, that is SA_robot, is contained within it as seen in the first column in Fig. S4. A drive forward area requires an intersection with the current robot as seen in the second column of Fig. S4, as other obstacles on this area may push the robot towards the edge. An avoid area requires either the intersection with the current robot or the intersection of a robot that has moved forward, that is, SA_robot_forward, visualized in third column of Fig. S3. In this work, we consider SA_robot_forward to be the geometric area of the robot that traversed in its heading $\vec{h}$ for two meters as this seemed a reasonable balance between not reacting too early and not too late. Note that taking too high values may result in the robot missing avoid areas. A no enter area is relevant when the

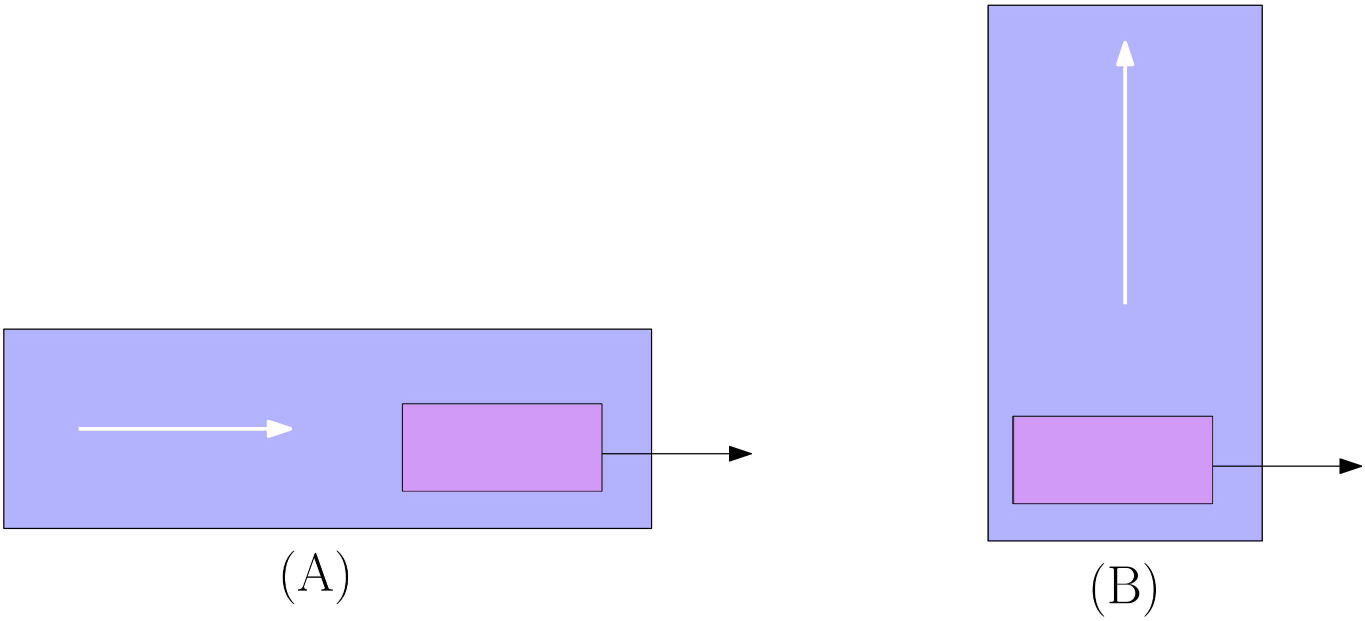

Figure S3. An example clarifying that an instantaneous lane change, as from (A) to (B), would result in a big angle difference between the robot (pink square) its current heading and the new lane direction vector.

Algorithm 1 Procedure of the implemented COP solver

procedure SOLVECOP
    //solve generated COP for most amount intentions
    while optimal input u not found **do**
        - obtain input u from COP-solver that satisfies all the constraints in 'list_constraints'
        if no input u can be found that satisfies all constraints **then**
            if all constraints in 'list_constraints' have highest intention **then**
                - optimal input $u = \vec{0}$
            else
                - remove the constraints belonging to the most inferior intention of 'list_constraints'
            end if
        else
            - optimal input u is the one that gives best objective score
        end if
    end while
end procedure

area around the robot intersects with it, that is, SA_robot_around. A circle with a radius of five meters is chosen to guarantee that all nearby no-enter areas are taken into consideration. It is necessary to also take the no-enter areas behind the robot into consideration as the robot could rotate into it with its back.

1.5 Semantic Behaviors

The Semantic Behaviors (SBs) are described in Tab. S2. The first column gives a short description, the second column the transformative Spatial Query (TSQ) with parameters in brackets, the third column the filtered_SA or SA on which the TSQ is applied, and the fourth column the Elementary Behavior (EB), with its parameterization in brackets, and intention knowledge.

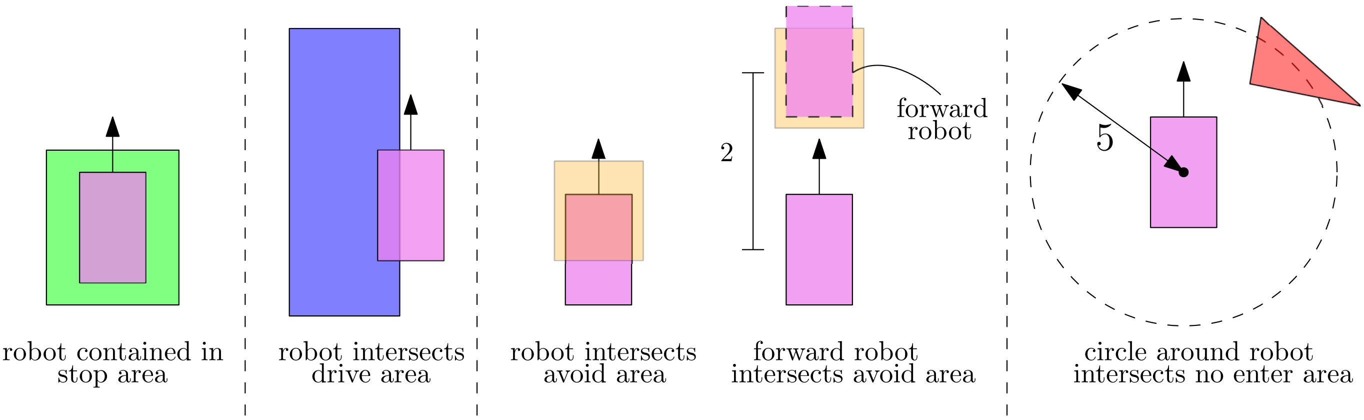

Figure S4. Visualizations depicting situations that would satisfy the monitor. The robot is visualized as a pink square with its heading depicted by the black arrow. The first column represent the satisfaction of the stop area, the second column the drive area, the third column the avoid area, and the fourth column the no enter area.

1.5.1 Non-continuous SBs

These SBs will only need to be solved at certain time instances and designs the static part of the behavior map. SBs 1-10 belong to this category. The first SB describes when the robot has reached its destination. The second SB chooses the lane that contains the goal and also intersects with the robot current position. One of the lanes L1-L7 will then be chosen at the bottom left of Fig. 10 in the main text. Each lane can be traversed in two directions, and the direction vector parameter in the fourth column is then one of those two directions that will direct the robot towards its goal. A translational speed limit of 0.7 m/s is chosen for the simulation and 0.5 m/s for the real world experiments. The relative higher speed limit for simulation is due to no risk of physically damaging the robot. The low speed limit overal is due to 1) the risk of physically damaging the robot, and/or 2) practical reasons to give the program more time to process the data as the frequency of the program varied between 1-10 Hz. The latter can be explained due to non-optimal coding and/or a python implementation with the shapely package performing the Transformative or Filtering Spatial Queries not fast enough. This could be problematic as the robot may displace a big distance before the monitor in the next program loop can check the relevant Behavior Areas at Alg. 3 in the main text. The robot may not react properly to the behavior map and in the worst case enter a no-enter area. The rotational speed limit is set at 40 deg/s for similar reasons of allowing the program to check the behavior map timely.

SB3-SB5 are designed to avoid being near hard objects and the Functional Area (FA) that may contain objects within it. We only generate the BAs of objects that are within the lane to prevent the bookkeeping of too much unrelated BAs. The geometric areas of walls and pillars are buffered with the postgis query 'ST_Buffer'. For walls a buffer value of 0.35 is chosen for the simulations and 0.6 for the real world experiments. For pillars a buffer value of 0.7 for simulations and 0.85 for real world experiments. The reason for the higher real-world value is due to the risk of physically damaging the robot. The overal low buffer value is chosen as otherwise the avoid area became too big such that the robot will often take those constraints of the avoid area into account as on the left of Fig. S5. As avoid areas are typically related to lower speed limits, the robot will drive unecessarily slow. This is one of the main tuning method of those avoid areas, that is, to choose a buffer value that would not unecessarily restrict the robots behavior in terms of velocity or position. The difference between pillars and walls buffer values is explained as the pillars can be present on any position in the lane whereas walls are only present at the sides of the lane. The

lowered translational speed of 0.2 m/s is due to us wanting the robot to slow down near those hard objects. The rotational speed was chosen as 20 deg/s in simulation and 40 deg/s in in real world experiments. This is due to us wanting to clarify the slow down behavior in simulations.

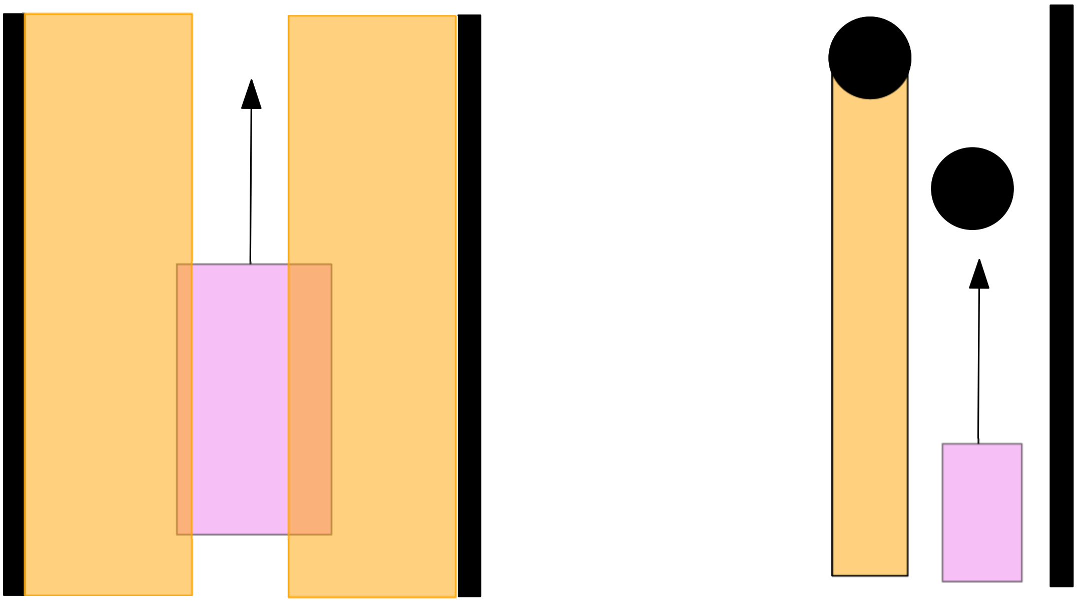

(A) too big avoid areas (B) too big length AreaInDirection

Figure S5. Visualization of too big avoid areas in (A) and too long avoid areas in (B). The black squares are walls and the black circles are pillars. The robot is visualized as a pink square with the direction implying its heading.

SB6 and SB7 are added as a premature measure to avoid these areas in case the avoid monitor does not look far enough ahead. We hence added a 3 meter avoid area on those objects with a direction vector that is opposite to the one of SB2 via a custom python function. These 3 meters showed proper behavior of precautiously avoiding the object. Defining bigger length values could result in the robot to not optimally use its space, e.g., as in the right of Fig. S5 where the robot would rather be more left to avoid the first pillar. SB8 is added to make sure that the robot stays in the lane. We create an avoid area around the lane with a buffer value of 2 meter via postgis queries 'ST_Buffer' and 'ST_difference'. It is not problematic for the robot to be on the edge of the lane, such that we do not impose reduced translational and rotational speed limits. SB9 and SB10 are added to prevent collisions with the walls and pillars.

1.5.2 Continuous SBs

These SBs will result in Behavior Areas (BAs) that need to be removed from the behavior map and are updated at every program loop as done via Alg. 2 in the main text. They are related to the robot perception, where the robot perception updates the semantic map at every loop of the program. This update occurs before Alg. 1 of the main text is activated. By updating the behavior map real-time, one can allow the robot to react to unforeseen circumstances, e.g., dynamic objects. SB11 and SB12 for example allows the robot to react to humans that are present on its lane. The resulting BAs will move according to the observed motion of the human. An example of the resulting BA is seen in Fig. 14 in the main text. The parameters that characterizes this shape is tuned by observing the robot its behavior whenever a human-robot passing

interaction occurs. We wanted the robot to react on time and also quickly choose the left or right side of the human, hence the long point shape. The width is 1) to make sure that the robot will stay far enough away from the human, and 2) to deal with uncertainties of the geometric position of the human detection algorithm. In this case a width is chosen that is a bit bigger than the human width as it may otherwise restrict the robot its position too much. The no-enter area is a smaller shape of the avoid area and more tailored to the geometric area occupied by the human. Note that we always assume any detected human to move away from the goal and towards the robot, as indicated by the direction parameter ”away from goal”. SB13 is introduced to deal with map uncertainties. It can occur that objects are not priorly put on the semantic map and can neither be detected via a camera. In this case we make use of the LRF, where we retrieve the coordinates of the laser points and check whether they are contained within a no-enter area on the behavior map. If no no-enter area can be found, then we classify the laser point as an unknown point ’U_LRF’. We then add these unknown points as new no-enter areas on the behavior map.

Table S2. Detailed description of the designed Semantic Behaviors. The first column describes the SB. The second column the Transformative Spatial Query with in brackets any optional parameters (Figure 4 in main text). The third column the argument on which the TSQ is applied. The fourth column the control knowledge where the first argument refers to the Elementary Behavior, with in brackets any optimal parameters (objectives/constraints of `EB_control` data structures and Table S1), and the second argument to the intention.

description	TSQ	FSQ/SA	control knowledge
1. stop at goal	EqualArea[]	Goal	1. stop[]<br>2. CompleteTask
2. drive to goal on lane	EqualArea[]	Intersects(Contains(Lane,Goal) ,Robot) = LGR	1. drive [v_{limit} = 0.7/0.5, ω_{limit} = 40, direction = to goal]<br>2. Progress
3. avoid walls on lane to goal	BufferArea [buffer = 0.35/0.6]	Intersects(Wall, LGR)	1. avoid [v_{limit} = 0.2 m/s, ω_{limit} = 20/40]<br>2. Safety
4. avoid pillars on lane to goal	BufferArea [buffer = 0.7/0.85]	Intersects(Wall, LGR)	1. avoid [v_{limit} = 0.2 m/s, ω_{limit} = 20/40]<br>2. Safety
5. avoid Functional Area (FA) on lane to goal	EqualArea []	Intersects(FA, LGR)	1. avoid [v_{limit} = 0.2 m/s, ω_{limit} = 20/40]<br>2. Safety
6. avoid driving into pillars	AreaInDirection [length = 3, direction = away goal]	Intersects(Pillar, LGR)	1. avoid [v_{limit} = 0.2 m/s, ω_{limit} = 20/40]<br>2. Safety
7. avoid driving into FA	AreaInDirection [length = 3, direction = away goal]	Intersects(FA, LGR)	1. avoid [v_{limit} = 0.2 m/s, ω_{limit} = 20/40]<br>2. Safety
8. stay in lane to goal	AroundArea [buffer = 2]	LGR	1. avoid [v_{limit} = 0.7/0.5 m/s, ω_{limit} = 40<br>2. Progress
9. No Collision Walls in lane	EqualArea []	Intersects(Wall, LGR)	1. no-enter []<br>2. NoDamage
10. No Collision Pillars in lane	EqualArea []	Intersects(Pillar, LGR)	1. no-enter []<br>2. NoDamage
11. pass humans respecting comfort distance	HumanInDirection [l1 = 1, l2 = 4, l3 = 4, width = 1, direction = away goal]	Intersects(human, LGR)	1. avoid [v_{limit} = 0.2 m/s, ω_{limit} = 20/40]<br>2. Safety
12. No Collision human in lane	HumanInDirection [l1=0.3, l2 = 0.3, l3 =0.3, width = 0.5, direction = away goal]	Intersects(human, LGR)	1. no-enter []<br>2. NoDamage
13. No Collision unknown laserpoints	EqualArea []	Intersects(U_LRF , LGR)	1. no-enter []<br>2. NoDamage
